# Identification of plasma biomarkers in a PTZ-induced Sudden Unexpected Death-like model through integrated proteomics and metabolomics methods

**DOI:** 10.3389/fmed.2026.1774546

**Published:** 2026-03-09

**Authors:** Gaolin Zheng, Xinyan Yang, Yinyu Chen, Peng Zhang, Qianyun Nie

**Affiliations:** 1 Key Laboratory of Tropical Translational Medicine of Ministry of Education, Department of Forensic Medicine, College of Basic Medical Sciences, Hainan Medical University, Haikou, China; 2 Hainan Academy of Medical Sciences, Hainan Medical University, Hainan, China; 3 Key Laboratory of Tropical Translational Medicine of Ministry of Education, Department of Pathology, College of Basic Medical Sciences, Hainan Medical University, Haikou, China

**Keywords:** biomarkers, metabolomics, plasma, proteomics, Sudden Unexpected Death in Epilepsy

## Abstract

**Introduction:**

Sudden Unexpected Death in Epilepsy (SUDEP) refers to the unexplained, sudden death of individuals with epilepsy, and its incidence is closely linked to the severity and duration of seizures. This study aimed to identify plasma biomarkers associated with SUDEP through a combined proteomics and metabolomics approach.

**Methods:**

A PTZ-induced seizure-related sudden death (SUDEP-like) paradigm was established in Sprague-Dawley (SD) rats via intraperitoneal injection of pentylenetetrazol (PTZ). Hippocampal histology was included to provide pathological context for plasma proteomic and metabolomic changes in the SUDEP-like model. Hippocampal tissue was extracted for hematoxylin and eosin (HE) staining to observe pathological changes. Blood was collected for plasma separation, followed by combined analysis using Data-dependent ion acquisition (dDIA) with Nanoflow Liquid Chromatography-Tandem Mass Spectrometry (nanoLC-MS/MS) for proteomics and Ultra-High Performance Liquid Chromatography-Quadrupole-Orbitrap Mass Spectrometry (UHPLC-QE-MS) for metabolomics.

**Results:**

Hematoxylin and eosin staining revealed a reduction in the number and morphological alterations of hippocampal neurons in SUDEP rats. Proteomic analysis identified 284 proteins that were significantly differentially expressed in the plasma of SUDEP rats. Key proteins such as mitogen-activated protein kinase 3 (Mapk3), protein BUD31 homolog (Bud31), heterogeneous nuclear ribonucleoprotein K (Hnrnpk), elongation factor 1-alpha (Eef1a1, Eef1a2), small ribosomal subunit proteins (Rps10, 11, 17, 20), and large ribosomal subunit proteins (Rpl23, 24, 38) were found to be highly associated with SUDEP and identified as potential plasma biomarkers. Metabolomic analysis revealed 565 metabolites that were significantly differentially expressed, leading to the identification of seven metabolic pathways potentially linked to the mechanisms of SUDEP. Combined analysis highlighted three pathways of significant association: β-alanine metabolism, sphingolipid metabolism, and pantothenate and Coenzyme A (CoA) biosynthesis. Furthermore, the integrated proteomic and metabolomic analysis highlighted 14 candidate plasma biomarkers. Among these, 11 molecules were identified in the metabolomic dataset, including sphinganine (DHS), phytosphingosine (PHS), sphingosine (SPH), sphingosine 1-phosphate (S1P), sphingomyelin (SM), glucosylceramide (GlcCer), β-alanine, carnosine, uracil, pantothenic acid, and L-histidine, whereas phosphatidylcholine:ceramide cholinephosphotransferase 2 (Sgms2), lysosomal acid glucosylceramidase precursor (Gba1), and aspartate 1-decarboxylase (Gadl1/Csad) were quantified in the plasma proteomic dataset and incorporated into the integrated pathway interpretation.

**Conclusion:**

These findings nominate candidate circulating signatures associated with seizure-related sudden death in this SUDEP-like paradigm. Translation to SUDEP risk stratification or forensic application requires independent validation in well-phenotyped human cohorts using standardized SUDEP classification.

## 1 Introduction

Sudden Unexpected Death in Epilepsy (SUDEP) refers to the unexplained sudden death of individuals with epilepsy, after excluding possibilities such as accidental injury due to loss of consciousness, including falls, drowning, or aspiration. The incidence of SUDEP is closely linked to the duration and severity of seizures. Previous studies have reported that the incidence of SUDEP among general epilepsy patients ranges from 0.9 to 2.3 per 1,000 person-years, while in patients with refractory epilepsy, the incidence rises to 1.1–5.9 per 1,000 person-years (1). For patients who are considering surgery or still experiencing seizures after surgery, the incidence further increases to 6.3–9.3 per 1,000 person-years (2).

The occurrence of SUDEP is likely a result of multiple contributing factors, with no single mechanism explaining all cases. Potential mechanisms underlying SUDEP may involve changes in respiratory function, cardiac function, and the autonomic nervous system (3–5). Seizures can lead to pulmonary dysfunction and suppression of the brainstem respiratory and arousal centers, resulting in insufficient ventilation (6). Some studies suggest that specific serotonin receptor antagonists may reduce the occurrence of desaturation of blood oxygen and respiratory arrest during seizures (7). Additionally, hypoxemia may increase the risk of arrhythmias, and in severe cases, lead to SUDEP (8). It has been proposed that stress-induced cardiomyopathy may be associated with the onset of SUDEP (9). Autonomic dysfunction in epilepsy patients, including reduced heart rate variability, may also be linked to increased mortality and higher rates of SUDEP (4). Furthermore, research has indicated that the occurrence of SUDEP is associated with certain genetic mutations, such as those in KCNQ1, KCNH2, SCN5A, HCN1-4, and others, which may increase the risk of sudden death in epilepsy patients (10).

Sudden Unexpected Death in Epilepsy holds significant forensic implications, particularly when the cause of death is unclear. Previous case studies have provided valuable practical insights into the relationship between hippocampal lesions and SUDEP. Hippocampal sclerosis (HS) is a common pathological feature of refractory temporal lobe epilepsy and has been described in a subset of SUDEP cases, but it is not a universal finding in SUDEP. Because limbic structures are involved in autonomic and respiratory regulation and seizures can trigger systemic stress responses and inflammation, hippocampal injury may be accompanied by measurable changes in plasma proteomic and metabolomic profiles. However, there remains controversy over its specific morphological markers, such as the dispersion of granule cells. Some studies have reported that 4% of post-mortem SUDEP cases exhibit dentate granule cell dispersion, yet these findings were not systematically evaluated through histological analysis (11). To address this, Somani et al. (12) conducted a study examining a large number of post-mortem cases from SUDEP and control groups to assess the potential of hippocampal granule cell dispersion and other morphological indicators, such as poor rotation, as biomarkers for SUDEP. However, the results failed to support the hypothesis that hippocampal morphological features, including excessive granule cell dispersion or hippocampal malformation associated with acute seizures and late mortality (HMAL), could serve as definitive biomarkers for SUDEP.

Since autopsies typically fail to reveal specific pathological changes, forensic experts often rely on the patient’s history of epilepsy and details of seizures before and after death to infer the likelihood of SUDEP. However, this inference lacks objective evidence and carries certain risks. In cases of epilepsy-related deaths with no witnesses or unclear seizure circumstances, it is difficult to confirm SUDEP through autopsy alone. As such, the forensic pathological diagnosis of SUDEP remains a challenging research issue in forensic science. Accurate and objective diagnosis of SUDEP is currently a key focus and challenge in forensic pathology.

As a result, SUDEP research faces challenges due to unclear mechanisms and the lack of objective diagnostic markers, prompting scholars to explore new research approaches. Proteomics, as a powerful research platform, has become a key tool for identifying disease-related proteins through mass spectrometry, providing critical insights into biomarker discovery and drug target identification (13, 14). Meanwhile, metabolomics, an emerging branch of systems biology, reveals disease mechanisms by analyzing dynamic changes in metabolites. It has been widely applied to the study of various complex diseases and has begun to uncover the role of immune and inflammatory regulation in epilepsy (15, 16). The combined analysis of omics data offers new avenues for exploring the mechanisms of SUDEP. Advances in technologies such as Liquid Chromatography-Tandem Mass Spectrometry (LC-MS/MS) and Ultra-High Performance Liquid Chromatography (UHPLC) have provided reliable tools for multi-omics research. Therefore, this study integrates plasma proteomics and metabolomics to characterize circulating molecular alterations associated with PTZ-induced seizure-related sudden death (SUDEP-like) in rats and to nominate candidate plasma biomarkers for subsequent validation.

## 2 Materials and methods

### 2.1 Chemicals and reagents

All formic acid, methanol (MeOH) and acetonitrile used in this experiment were of HPLC grade, and obtained from Sigma-Aldrich (St. Louis, MO, USA). Protein quantitation kit was purchased from Applied Biosystems (Foster City, CA, USA). Ultrapure water used in this experiment was prepared using a Milli-Q water purification system (Millipore, Bedford, MA, USA).

### 2.2 Animals and ethics statement

This study utilized 32 healthy adult male Sprague-Dawley (SD) rats, aged 8 weeks, with a weight range of 250–300 g. One week prior to the experiment, the rats were housed in a standard environment with a temperature of 22 °C ± 2 °C, relative humidity of 60% ± 5%, and a 12-h light/dark cycle. They were provided with ample water and standard rodent chow. All experimental procedures and animal care were conducted in strict accordance with relevant guidelines and approved by the Animal Ethics Committee of Hainan Medical University (approval number: HYLL-2021-303), following all applicable animal welfare regulations.

### 2.3 Establishment of the PTZ-induced seizure-related sudden death (SUDEP-like) paradigm and sample collection

For the SUDEP-like group (PTZ-induced seizure-related sudden death), SD rats were administered a 60 mg/kg intraperitoneal injection of 1% pentylenetetrazol (PTZ) solution between 8:00 and 10:00 AM, with injections given every other day until sudden death occurred; hereafter termed “SUDEP-like” to denote a surrogate phenotype rather than definitive clinical SUDEP. The control group received an equivalent volume of saline solution at the same time intervals, and were euthanized via anesthesia once the death time of the experimental group was reached. Prior to each injection, the rats were weighed and the dose adjusted accordingly. Seizure onset and severity were recorded within 1 h following injection. Continuous EEG, ECG, respiratory, or oximetry recordings were not obtained, and seizure severity was assessed based on behavioral manifestations. Blood was collected from the right ventricle into heparinized EP tubes immediately after confirmation of death in the acute SUDEP group or immediately after anesthetic euthanasia in the control group. Plasma was separated by centrifugation at 3500 rpm for 20 min at 4 °C, and the supernatant was stored at −80 °C for subsequent omics analysis. The whole brain was quickly removed, rinsed with pre-chilled phosphate-buffered saline (PBS), and the hippocampal tissue was isolated on ice. Two samples were randomly selected and fixed in 10% neutral formalin for pathological analysis. All procedures were performed under standardized conditions to ensure data reliability. The experimental timeline is summarized in Supplementary Figure 1.

### 2.4 Histopathological examination

Two rats from each group were randomly selected for histopathological examination. The hippocampal tissues were fixed in paraffin and sectioned into 5 μm slices. Hematoxylin and eosin (HE) staining was performed, and the tissue pathological changes were observed under an optical microscope. Given the limited sample size (*n* = 2 per group), the histopathological observations were intended to be qualitative and descriptive and were not subjected to quantitative stereology or statistical testing.

### 2.5 Proteomics analysis

#### 2.5.1 Sample preparation and dDIA-LC-MS/MS proteomics

For proteomics analysis, 6 frozen plasma samples from each group were selected. After magnetic bead-based depletion of high-abundance proteins, the samples were reduced with dithiothreitol (DTT), digested with trypsin, desalted, and concentrated. The resulting peptides were then subjected to nano-UPLC-DIA mass spectrometry analysis. A total of 500 ng of peptides were separated using a nano-UPLC system (EASY-Spray column) with a gradient of 0.1% formic acid/acetonitrile, followed by Data-Independent Acquisition (DIA) mass spectrometry analysis (MS1 scan range: 380–980 m/z, MS2 scan range: 150–2000 m/z, NCE 25%).

#### 2.5.2 Data processing and pathway and interaction network analysis

Raw mass spectrometry files were imported into DIA-NN (version 1.8.1) software for database search, followed by qualitative analysis. Prior to screening for differentially expressed proteins, the data were normalized. Differentially expressed proteins were selected based on the following criteria: *P*-value < 0.05 from Student’s *t*-test, and Fold Change (FC) < 0.67 or FC > 1.5. The identified protein data were subjected to functional enrichment analysis using Gene Ontology (GO) tools^1^ to explore the distribution and roles of these proteins in molecular function (MF), biological processes (BP), and cellular components (CC). Additionally, protein-protein interaction (PPI) data were retrieved from the STRING database (v11)^2^. After data extraction, a PPI network was constructed using Cytoscape v3.9.1 software, and clustering analysis of the differentially expressed proteins was performed using R programming.

### 2.6 Metabolomics analysis

#### 2.6.1 Sample preparation and UHPLC-QE-MS analysis

For metabolomics analysis, 8 frozen plasma samples from each group were selected. A 100 μL aliquot of each sample was extracted with a methanol-acetonitrile (1:1, containing internal standards) mixture, followed by low-temperature protein precipitation. After centrifugation, the supernatant was collected and used to prepare quality control (QC) samples for metabolomics analysis. Metabolites were separated using a BEH Amide column (2.1 mm × 50 mm), with a mobile phase consisting of ammonium acetate-acetonitrile at pH 9.75. The analysis was performed using an Orbitrap Exploris 120 mass spectrometer in IDA mode (resolution 60 k/15 k, stepped collision energy).

#### 2.6.2 Data processing and analysis

Raw data were converted into mzXML format using ProteoWizard 3.0 software and metabolite identification was carried out using the R package (version 4.0.3) with the BiotreeDB (V3.0) database. After data acquisition, SIMCA software (V18.0.1) was used for logarithmic transformation and UV scaling to eliminate dimensional discrepancies and enhance model stability. Principal component analysis (PCA) and Orthogonal Partial Least Squares Discriminant Analysis (OPLS-DA) were employed to discriminate between plasma samples from the acute SUDEP group and the control group. Model quality was assessed using 7-fold cross-validation, and model validity was evaluated through R^2^Y (the model’s explanatory power for the classification variable Y) and Q^2^ (the model’s predictive ability). The model’s effectiveness was further validated by permutation testing (200 permutations), in which the classification variable Y’s order was randomly shuffled, and random Q^2^ values were calculated. Based on the results from both univariate and multivariate statistical analyses, significantly altered metabolites were selected. The criteria for selecting significantly altered variables were: (1) Variable Importance in Projection (VIP) > 1 in the first principal component of the OPLS-DA model; (2) FC < 0.67 or FC > 1.5 between the acute SUDEP group and the control group; (3) *P*-value < 0.05 from Student’s *t*-test. Pathway analysis and visualization of significantly altered metabolites were performed based on the KEGG database using R packages (KEGG graph, ggplot2, and treemap) to uncover the regulatory mechanisms of metabolites under experimental conditions, analyze the metabolic changes, and explore their potential roles in biological pathways.

### 2.7 Integrated analysis of proteomics and metabolomics

To further investigate the relationship between differentially expressed proteins and metabolites, this study performed an integrated analysis combining proteomics and metabolomics. First, we organized the upregulation and downregulation of differentially expressed metabolites and proteins from the proteomics and metabolomics datasets, selecting those with significant differences (FC > 1.5 or <0.67, *P*-value < 0.05). Then, the gene identifiers (such as KEGG ID) of these differentially expressed metabolites and proteins were imported into the KEGG Pathway database,^3^ and mapped to all relevant pathways in the species *Rattus norvegicus* (rat). Using the KEGG Mapper tool, we generated an integrated pathway map for the metabolites and proteins, identifying key metabolic pathways associated with epilepsy. Subsequently, metabolic pathways were selected based on fold enrichment and a corrected *P*-value < 0.05.

### 2.8 Statistical analysis

All protein and metabolomics data were analyzed using SPSS 21.0 statistical software. The normality of the data distribution was assessed using the Shapiro-Wilk test. ANOVA was used to determine significant differences between multiple groups, followed by *post hoc* testing with Tukey’s HSD test. A two-tailed test was applied, and a significance level of *P* < 0.05 was used to assess statistical significance.

## 3 Results

### 3.1 Histopathological examination of the hippocampus

**FIGURE 1:**
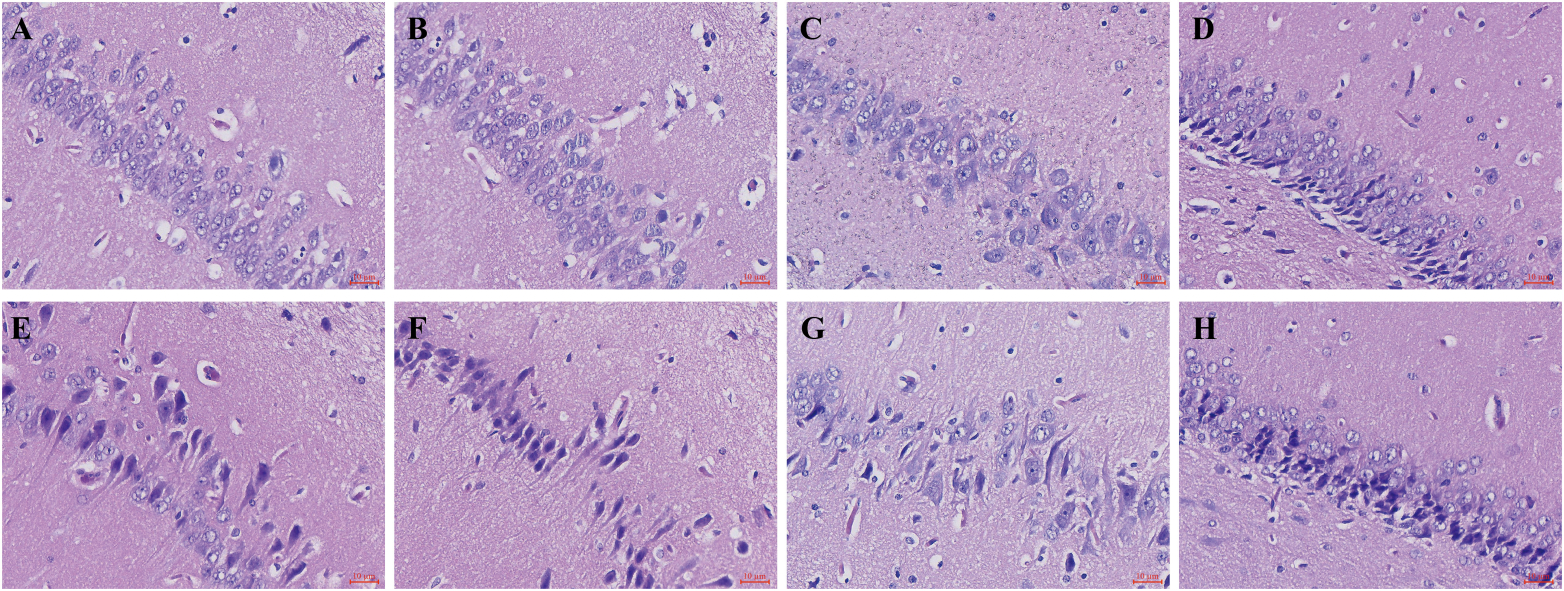
Histological observation of the hippocampal tissue in SD rats with acute SUDEP by HE staining (×200). **(A,B,E,F)** CA1 region, **(C,G)** CA3 region, **(D,H)** DG region.

In the control group (Figures 1A–D), the neurons in the CA1, CA3 (cornu ammonis 1 and 3), and dentate gyrus (DG) regions were neatly arranged, with normal cell morphology, clear cell nuclei, and moderate intercellular spacing. The overall neuronal cytoarchitecture appeared preserved, with no obvious signs of neuronal deformation, vacuolation, or damage. In the acute SUDEP group (Figures 1E–H), neurons in the CA1, CA3, and DG regions appeared reduced in number, with disordered cell arrangement. Many cells showed signs of deformation and atrophy, with irregularly shaped nuclei. Some cells exhibited vacuolation or were absent.

### 3.2 Plasma proteomics identifies differentially expressed proteins

#### 3.2.1 Selected differential proteins

**FIGURE 2:**
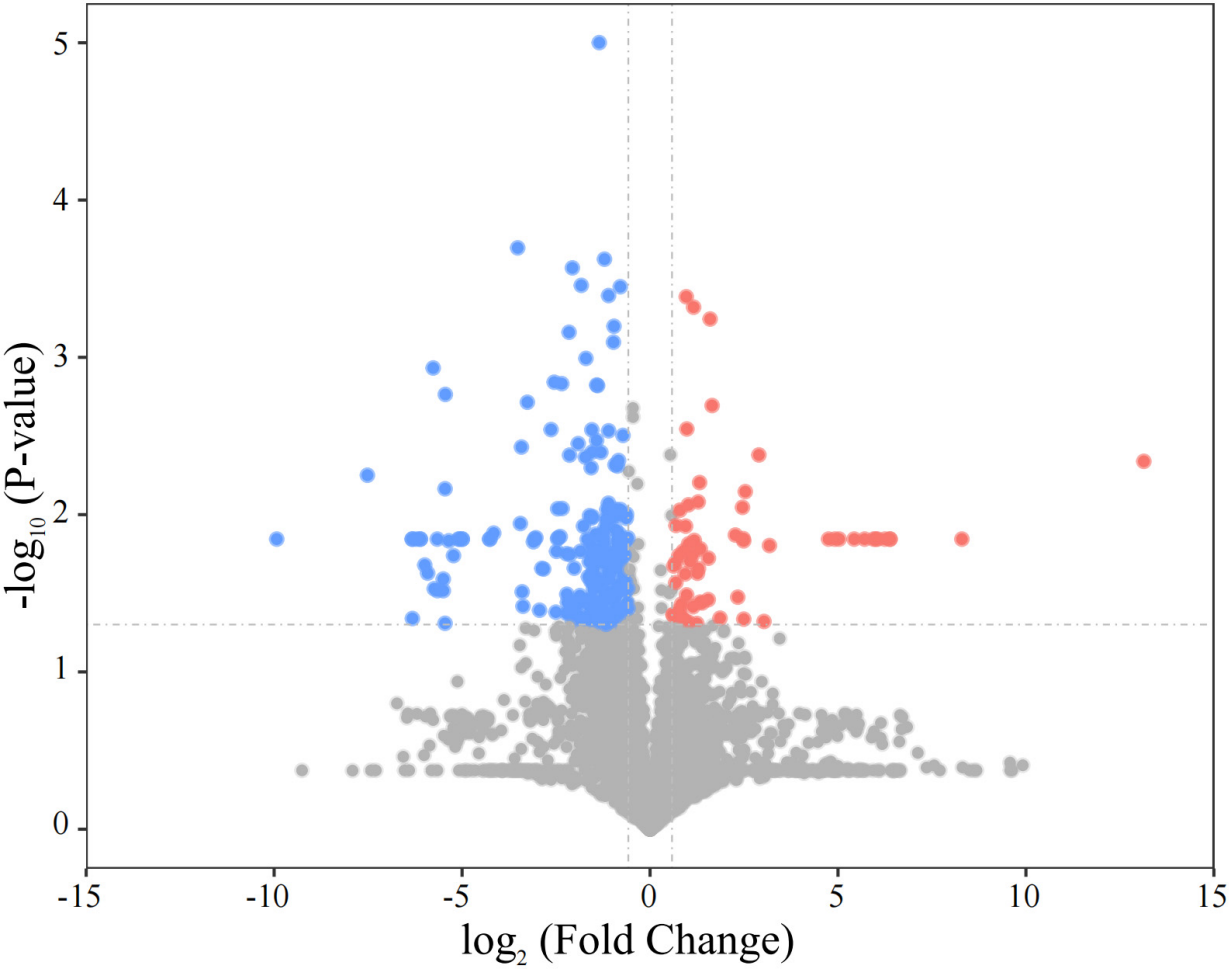
Volcano plot showing the changes in plasma protein expression in SD rats following acute SUDEP. Each point represents a detected protein, with scatter point colors indicating the final selection results. Red represents significantly upregulated proteins, blue represents significantly downregulated proteins, and gray represents proteins with non-significant differences.

In this study, differential proteins in the plasma of SD rats after acute SUDEP were analyzed using dDIA combined with nanoLC-MS/MS technology. Protein database searches were performed using DIA-NN (version 1.8.1) software. Both qualitative and quantitative analyses identified a total of 4,242 proteins. Statistical analysis was performed to identify differentially expressed proteins, with the selection criteria being *P*-value < 0.05 from Student’s *t*-test, and FC < 0.67 or FC > 1.5. A total of 284 proteins were identified as differentially expressed (Figure 2), including 65 upregulated proteins and 219 downregulated proteins (Supplementary Table 1).

#### 3.2.2 GO analysis

In this study, GO analysis was performed to conduct functional enrichment of the differentially expressed proteins. The results indicated that the identified proteins exhibited a broad distribution and diverse functions in cells, involving multiple biological processes (Supplementary Figure 2). In terms of CC, these proteins were predominantly located in cellular stress granules, spliceosome complexes, cytoplasmic ribonucleoprotein particles, ribonucleoprotein complexes, axons, supramolecular complexes, cytoplasm, and organelles. MF analysis revealed that these proteins possessed various functions, including annealing activity, mRNA circular structure binding, mRNA 3’-UTR binding, calcium-dependent protein binding, single-stranded RNA binding, mRNA binding, RNA binding, and protein binding. BP analysis showed that these proteins were involved in biological processes such as mRNA splicing via the spliceosome, RNA splicing using amino adenosine as a nucleophile through transacylation reactions, RNA splicing, mRNA processing, RNA processing, cell localization, cellular component organization, and biosynthesis. GO analysis results suggest that the study of differential protein expression in the plasma of SD rats after SUDEP-like fatal seizure events, based on dDIA combined with nanoLC-MS/MS technology, is a feasible approach for characterizing associated pathophysiological alterations.

#### 3.2.3 PPI network analysis

**FIGURE 3:**
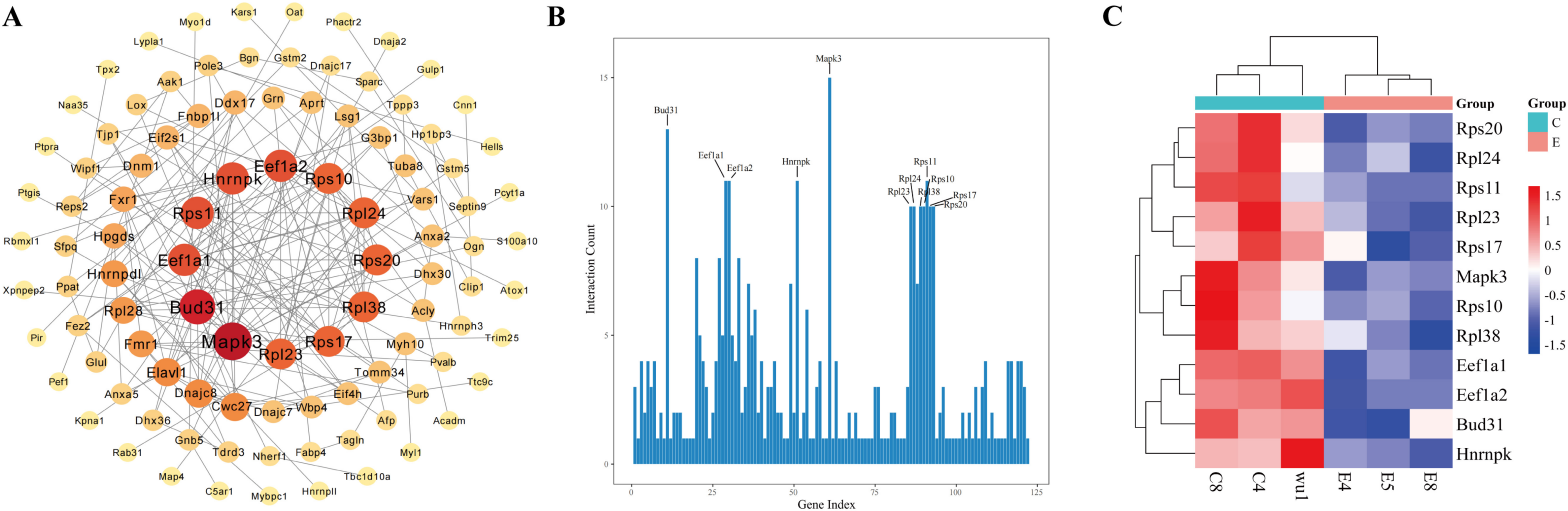
Interaction network and clustering analysis of DEPs in the plasma of SUDEP rats. **(A)** PPI network of DEPs in the plasma of SD rats after acute SUDEP. The size and color of the nodes represent their contribution to the network, with larger nodes indicating stronger contributions. The color and thickness of the edges represent the type and strength of interactions between nodes, with thicker edges indicating stronger interactions. **(B)** Histogram of interaction connectivity of differentially expressed proteins in the plasma following acute SUDEP. **(C)** Clustering analysis of the selected differential proteins.

To elucidate the core regulatory genes, a PPI analysis was conducted. The PPI network illustrated the interactions between differentially expressed proteins (DEPs) in the acute SUDEP group (Figure 3A). Based on the PPI network topology of the differentially expressed proteins, 12 core proteins with the highest network connectivity were selected for subsequent visualization and discriminatory performance analysis (within this dataset). The network connectivity analysis of the acute SUDEP group compared to the control group revealed several key proteins, including mitogen-activated protein kinase 3 (Mapk3), protein BUD31 homolog (Bud31), heterogeneous nuclear ribonucleoprotein K (Hnrnpk), elongation factor 1-alpha (Eef1a1, Eef1a2), small ribosomal subunit proteins (Rps10, Rps11, Rps17, Rps20), and large ribosomal subunit proteins (Rpl23, Rpl24, Rpl38) (Figure 3B). Among these, Mapk3 had the highest number of interacting genes/proteins, with 15 interactions identified. Additionally, hierarchical clustering analysis of the selected differential proteins indicated that these 12 proteins exhibited significant expression differences, all of which were downregulated (Figure 3C).

### 3.3 Plasma metabolomics analysis in SD rats with acute SUDEP

#### 3.3.1 Selected differential metabolites

**FIGURE 4:**
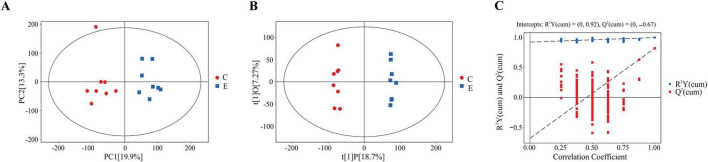
Multivariate statistical analysis of the plasma metabolomics in SUDEP rats: PCA, OPLS-DA, and model validation. **(A)** PCA score plot comparing the acute SUDEP group and control group. PC[1] and PC[2] represent the first and second principal component scores, respectively. Each scatter point corresponds to a sample, and the distance between sample points reflects the similarity of metabolic compositions. Most sample points are distributed within the 95% confidence interval. **(B)** OPLS-DA score plot comparing the acute SUDEP group and control group. t[1]P represents the predicted principal component score for the first principal component, reflecting inter-group differences; t[1]O represents the orthogonal component score, reflecting intra-group differences. **(C)** OPLS-DA model permutation test result for the acute SUDEP and control groups. The *x*-axis represents the permutation retention degree. The *y*-axis represents the R^2^Y or Q^2^ values. Blue circular points represent R^2^Y values obtained through permutation tests, and red square points represent Q^2^ values. The two dashed lines represent the regression lines for R^2^Y and Q^2^.

**FIGURE 5:**
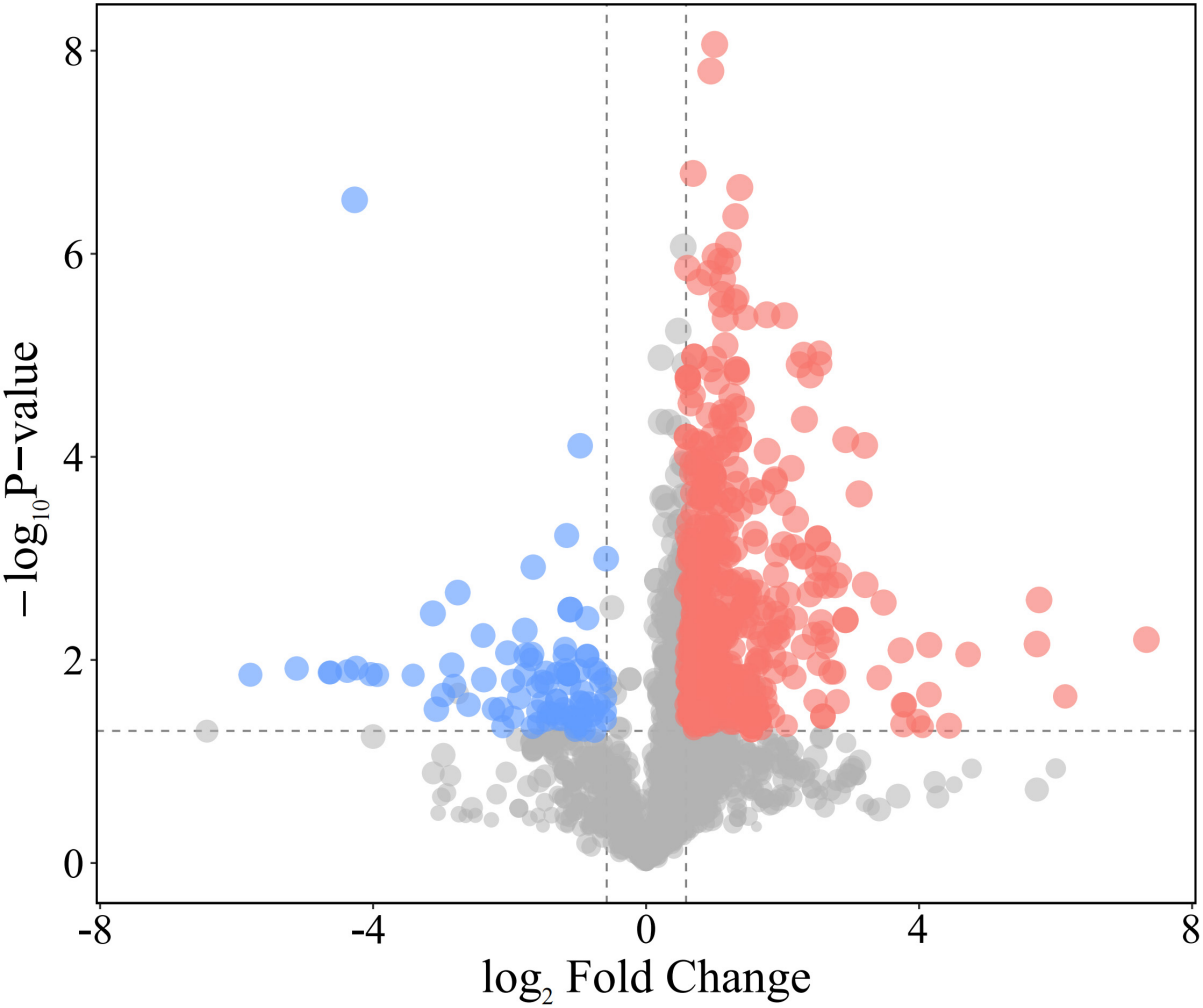
Volcano plot of the differential metabolites between the acute SUDEP and control groups. Each point represents a metabolite. Red represents significantly upregulated metabolites, blue represents significantly downregulated metabolites, and gray represents metabolites with no significant differences.

In this study, the metabolic profiles of plasma samples from acute SUDEP rats and control rats were analyzed using a UHPLC-QE-MS system in both positive and negative ion scanning modes. The relative standard deviation (RSD) values of QC samples confirmed the stability and applicability of the metabolomics analysis system, indicating that the method was suitable for subsequent analysis (Supplementary Table 2). Principal component analysis (PCA) was performed to assess the differences between the control and acute SUDEP groups, revealing significant differences between the plasma samples of acute SUDEP rats and control rats. This suggested that the plasma metabolism in the acute SUDEP rats was significantly disturbed (Figure 4A). To further elucidate the differential variables between the groups, Orthogonal Partial Least Squares Discriminant Analysis (OPLS-DA) was applied (Figure 4B) The OPLS-DA model parameters were R^2^Y = (0, 0.92), and Q^2^ = (0, −0.67). A permutation test (*n* = 200) was performed for model validation (Figure 4C). A total of 6,031 metabolites were quantitatively analyzed. Differential metabolites were selected based on the criteria of FC > 1.5 or <0.67, *P*-value < 0.05, and VIP > 1. A total of 565 metabolites were identified as differentially expressed (Figure 5), of which 475 were upregulated and 90 were downregulated (Supplementary Table 3).

#### 3.3.2 Metabolic pathway analysis

**FIGURE 6:**
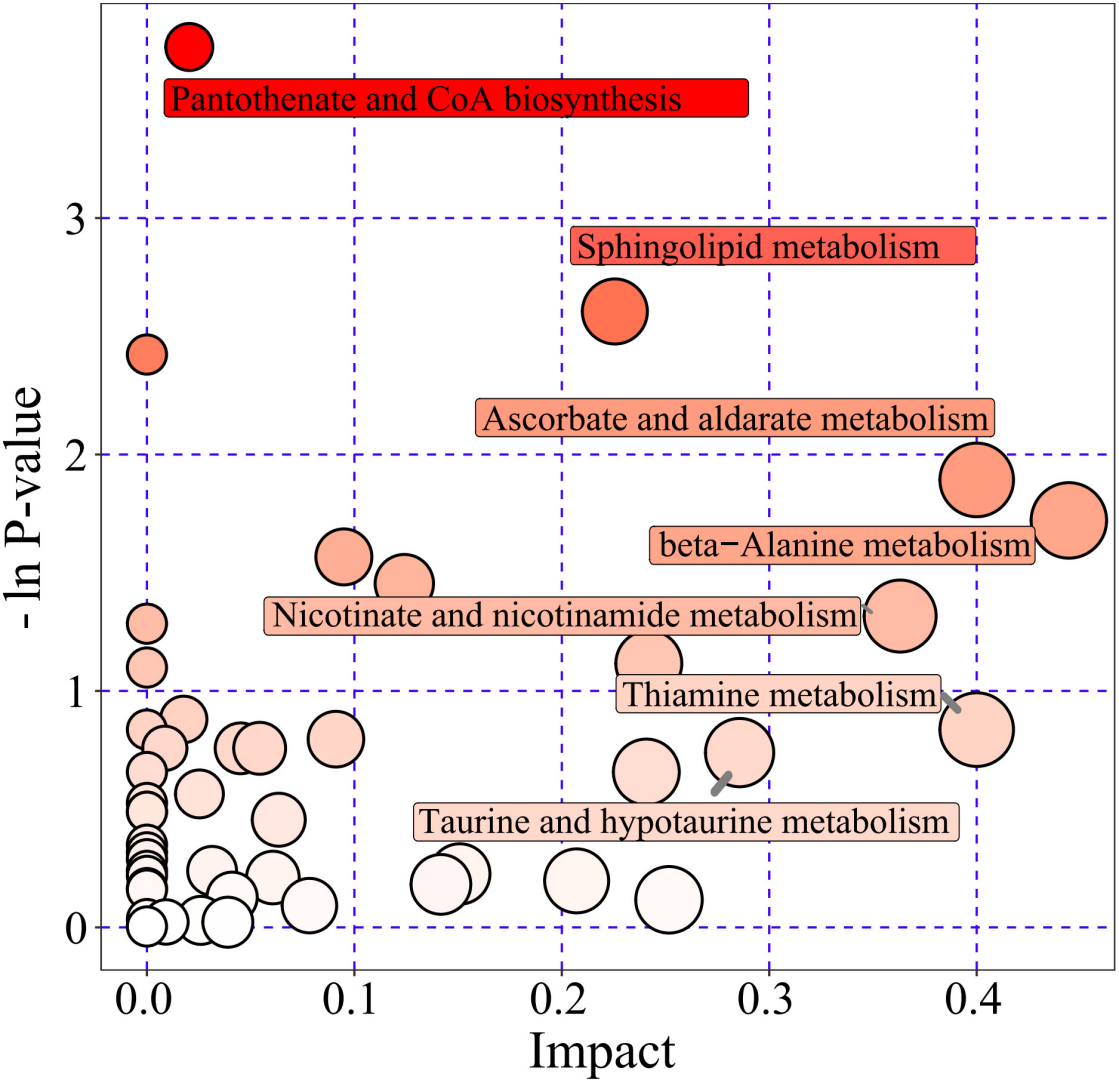
Metabolic pathway analysis for the acute SUDEP group. Each bubble represents a metabolic pathway, with the *x*-axis and bubble size indicating the pathway’s impact factor in topological analysis–the larger the bubble, the greater the impact factor. The *y*-axis and bubble color represent the enrichment analysis’s *P*-value [log-transformed as -ln(p)], with darker colors indicating smaller *P*-values and more significant enrichment.

To comprehensively understand the changes in metabolic pathways, R packages (KEGGgraph, ggplot2, and treemap) were used for further analysis of the metabolites that exhibited significant changes. This analysis aimed to identify the most relevant pathways potentially associated with seizure-related sudden death (SUDEP-like) in this paradigm. The results indicated that 46 metabolic pathways were affected in the acute SUDEP group. Among these, seven metabolic pathways were significantly disrupted, including sphingolipid metabolism, β-alanine metabolism, vitamin C and aldaric acid salt metabolism, thiamine metabolism, niacin and nicotinamide metabolism, taurine and hypotaurine metabolism, and pantothenate and Coenzyme A (CoA) biosynthesis (Figure 6). These pathways were highlighted due to their high impact values and -ln(p) values, suggesting a potential link to seizure-related sudden death (SUDEP-like) in this paradigm.

### 3.4 Integrated analysis of proteomics and metabolomics

**FIGURE 7:**
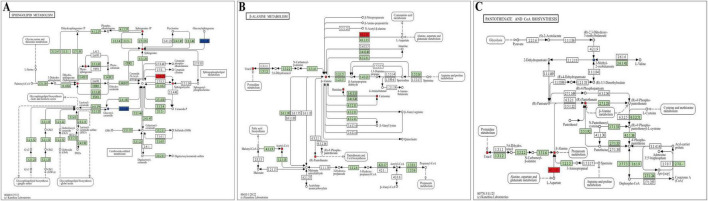
Differential metabolic pathways and molecular expression changes associated with SUDEP. **(A)** Sphingolipid metabolism pathway. **(B)** β-Alanine metabolism pathway. **(C)** Pantothenate and CoA biosynthesis pathway. Red represents upregulated metabolites or proteins, while blue represents downregulated ones. Squares represent proteins, and circles represent metabolites.

An integrated analysis of differential proteins and metabolites was performed to explore the protein-metabolite relationships in rats with SUDEP. Figure 6 summarizes pathway enrichment based on metabolomics alone. For the integrated analysis, differential proteins and differential metabolites were jointly mapped to pathways, and pathways were retained only when they remained significant under the corrected *P*-value threshold (corrected *P*-value < 0.05). The results showed that three metabolic pathways–sphingolipid metabolism (Figure 7A), β-alanine metabolism (Figure 7B), and pantothenate and CoA biosynthesis (Figure 7C)–were significantly disrupted (Table 1).

**TABLE 1:** Integrated analysis of pathways from proteomics and metabolomics.

No.Pathway name	Fold enrichment	*Q*-value	Class	Names
1Beta-Alanine metabolism rno00410	6.098	0.003	Metabolite	↑C00386 Carnosine
			Metabolite	↑C00106 Uracil
			Metabolite	↑C00864 Pantothenate
			Metabolite	↑C00099 beta-Alanine
			Metabolite	↑C00135 L-Histidine
			Metabolite	↑C00024 Acetyl-CoA
			Protein	↑Csad: aspartate 1-decarboxylase
2Sphingolipid metabolism rno00600	5.445	0.003	Metabolite	↑C00836 Sphinganine
			Metabolite	↑C12144 Phytosphingosine
			Metabolite	↑C00319 Sphingosine
			Metabolite	↑C06124 Sphingosine 1-phosphate
			Metabolite	↑C00550 Sphingomyelin
			Metabolite	↓C01190 Glucosylceramide
			Protein	↑Sgms2: phosphatidylcholine:ceramide cholinephosphotransferase 2
			Protein	↓Gba1: lysosomal acid glucosylceramidase precursor
3Pantothenate and CoA biosynthesis rno00770	5.445	0.025	Metabolite	↑C00106 Uracil
			Metabolite	↑C00864 Pantothenate
			Metabolite	↑C00099 beta-Alanine
			Metabolite	↓C00141 3-Methyl-2-oxobutanoic acid
			Protein	↑Csad: aspartate 1-decarboxylase

↑ Indicates upregulation; ↓ Indicates downregulation.

## 4 Discussion

### 4.1 Histopathological changes in the hippocampal tissue

The hippocampus is frequently involved in epilepsy, and hippocampal sclerosis is a common pathological finding in refractory temporal lobe epilepsy (17). Because limbic structures have connections with autonomic and respiratory networks, hippocampal injury has been discussed in relation to SUDEP in a subset of cases (18–22).

In this study, HE staining showed a reduction in hippocampal neurons together with morphological injury and disorganization in the acute SUDEP model, and neuronal atrophy and nuclear pyknosis were observed in the CA1, CA3 and DG regions (23). These observations provide pathological context for the plasma proteomic and metabolomic alterations, but they should be interpreted as associative rather than direct evidence for specific central mechanisms (24). During acute seizures, hippocampal neurons become excessively excited, potentially causing a large influx of calcium ions, which ultimately leads to cellular damage and death. This widespread damage to hippocampal neurons may impair their ability to regulate autonomic functions, thereby potentially triggering sudden death. These findings highlight the progressive nature of hippocampal degeneration in epilepsy patients and its correlation with cognitive deficits.

### 4.2 Potential SUDEP biomarkers identified based on PPI analysis

Based on PPI analysis, 12 proteins (Mapk3, Bud31, Eef1a1, Eef1a2, Hnrnpk, Rps10, Rps11, Rps17, Rps20, Rpl23, Rpl24, and Rpl38) were prioritized for discussion as candidate plasma biomarkers in the acute SUDEP model.

#### 4.2.1 Mapk3

Mitogen-activated protein kinase 3 (ERK1) is a core component of MAPK/ERK signaling and participates in neuronal stress responses and synaptic regulation (25–30). Evidence from network-based analyses has implicated Mapk3 in epilepsy-related signaling pathways (31). In this study, Mapk3 was downregulated in plasma after acute SUDEP, suggesting altered MAPK/ERK pathway activity under severe seizure burden and associated systemic stress (32).

#### 4.2.2 Bud31

Protein BUD31 homolog participates in spliceosome assembly and RNA splicing (33–35). Haploinsufficiency of Bud31 and dysfunction of related splicing factors have been associated with neurodevelopmental phenotypes including epilepsy (36–39). In this study, Bud31 was downregulated in plasma after acute SUDEP, supporting a potential association between RNA-processing dysregulation and the molecular response to severe seizures.

#### 4.2.3 Elongation factor 1 (Eef1a1, Eef1a2)

Eukaryotic translation elongation factor 1A is encoded by two isoforms, Eef1a1 and Eef1a2; Eef1a2 is enriched in neural tissues (40, 41). Genetic and transcriptomic evidence links Eef1a1 and Eef1a2 to neurodevelopmental phenotypes including epilepsy, and reduced expression has been reported in epileptic brain tissue (42, 43). Here, both Eef1a1 and Eef1a2 were downregulated in plasma after acute SUDEP, indicating an altered translation-related signature associated with severe seizures.

#### 4.2.4 Hnrnpk

Heterogeneous nuclear ribonucleoprotein K is an RNA-binding protein involved in RNA processing and post-transcriptional regulation (44). Variants affecting Hnrnpk and other hnRNP family members have been associated with neurodevelopmental and neurodegenerative phenotypes, including epilepsy (45–47). In this study, Hnrnpk was downregulated in plasma after acute SUDEP, suggesting that altered RNA-processing pathways may accompany severe seizure-related events.

#### 4.2.5 Ribosomal proteins

Ribosomal proteins are essential for translation and contribute to post-transcriptional regulation in the nervous system (48). Alterations in ribosome-related factors have been reported in epilepsy-relevant pathologies such as focal cortical dysplasia (49). In this study, several small- and large-subunit ribosomal proteins (Rps10, Rps11, Rps17, Rps20; Rpl23, Rpl24, Rpl38) were downregulated in plasma after acute SUDEP, consistent with a disturbance of protein synthesis related pathways under severe seizure burden.

### 4.3 Potential SUDEP biomarkers identified based on metabolic pathway analysis

This study employed untargeted UHPLC-QE-MS metabolomics to profile plasma from the acute SUDEP model and controls. Seven pathways were enriched by KEGG analysis. Integrated analysis with the proteomic dataset prioritized three pathways, namely sphingolipid metabolism, beta-alanine metabolism, and pantothenate and CoA biosynthesis. Within sphingolipid metabolism, sphingolipid metabolites including DHS, PHS, SPH, S1P, and SM were increased, whereas GlcCer was decreased. Within beta-alanine metabolism and pantothenate and CoA biosynthesis, beta-alanine, carnosine, uracil, pantothenic acid, and L-histidine were increased, whereas 3M2OB was decreased. Enzymes mapped to these pathways (e.g., SGMS2, GBA1, and GADL1 or CSAD) were identified from the proteomic dataset during integration.

#### 4.3.1 Sphingolipid metabolism

Sphingolipids are abundant in the nervous system and participate in membrane organization and signaling (50, 51). In this study, integrated analysis highlighted sphingolipid metabolism, with increased DHS, PHS, SPH, S1P, and SM and decreased GlcCer. Prior studies have linked disturbed sphingolipid metabolism and related enzymes to epilepsy and neuronal injury (52–63). The present findings indicate that sphingolipid-related changes in plasma accompany the acute SUDEP model and may support biomarker discovery at the pathway level, while mechanistic attribution requires further validation.

#### 4.3.2 β-alanine metabolism and pantothenate and CoA biosynthesis

Beta-alanine metabolism and pantothenate and CoA biosynthesis are metabolically connected and contribute to cellular energy homeostasis and stress responses (64–67). In this study, beta-alanine, carnosine, uracil, pantothenic acid, and L-histidine were increased and 3M2OB was decreased, and these features mapped to both pathways in the integrated analysis. Prior work supports roles of beta-alanine-related metabolites and carnosine in modulating excitability and oxidative stress (65–68). These pathway-level alterations suggest a systemic metabolic response in the SUDEP-like paradigm; future studies incorporating respiratory and electrophysiological monitoring may help to determine their relationship to peri-ictal cardiorespiratory dysfunction.

However, because plasma molecular profiles are strongly influenced by terminal physiological conditions and because continuous EEG, ECG, respiratory, or oximetry recordings were not obtained (see Section “2.3 Establishment of the PTZ-induced seizure-related sudden death (SUDEP-like) paradigm and sample collection”), these pathway-level signals should be interpreted as circulating correlates in this SUDEP-like paradigm and with consideration of peri-mortem effects.

## 5 Conclusion

Through this study, we have identified potential plasma biomarkers associated with PTZ-induced seizure-related sudden death (SUDEP-like), such as Bud31, Eef1a1, Eef1a2, and β-alanine. These biomarkers open up new avenues for hypothesis-driven risk assessment, early warning, therapeutic interventions, and biomarker-based discrimination in this SUDEP-like paradigm, rather than definitive diagnostic identification in SUDEP. However, the results of this study are based on animal model experiments, and the expression patterns of the selected differential proteins and metabolites in human epilepsy and SUDEP cases require further validation. Future research should focus on optimizing plasma biomarker screening systems to provide supporting evidence for forensic laboratories, aiding in the identification of high-risk epilepsy patients, and guiding clinical strategies for effective prevention and treatment to reduce the risk of sudden death. Furthermore, exploring the mechanisms of these biomarkers in the pathogenesis of epilepsy and SUDEP, particularly how they regulate relevant metabolic pathways in relation to seizure-related sudden death (SUDEP-like), will be crucial for early disease intervention. With the development of liquid biopsy and high-throughput analysis technologies, integrating multi-omics data will help comprehensively uncover the mechanisms of epilepsy and SUDEP-related phenotypes, providing scientific evidence for the development of personalized treatment plans.

## Data Availability

The datasets presented in this study can be found in online repositories. The names of the repository/repositories and accession number(s) can be found in the article/Supplementary Material.

## Footnotes

1
http://www.geneontology.org/

2
https://string-db.org/

3
http://www.kegg.jp/kegg/pathway.html
