# Supplementary material for: Identification of plasma biomarkers in a PTZ-induced Sudden Unexpected Death-like model through integrated proteomics and metabolomics methods
Source: Front Med (Lausanne). 2026 Mar 9;13:1774546. doi: 10.3389/fmed.2026.1774546 (PMC13006514; doi:10.3389/fmed.2026.1774546)
Supplement: Supplementary file 1 [file Data_Sheet_1.docx]

**Supporting information**

**Identification of Plasma Biomarkers in a PTZ-Induced Sudden Unexpected Death-Like Model through Integrated Proteomics and Metabolomics Methods**

Gaolin Zheng^1†^, Xinyan Yang1^†^, Yinyu Chen^1†^, Peng Zhang^1,2*^, Qianyun Nie^1,2**^

^1^ Department of Forensic Medicine & Key Laboratory of Tropical Translational Medicine of Ministry of Education, college of basic medical sciences, Hainan Medical University, Haikou 571199, China.

^2^ Department of Pathology & Key Laboratory of Tropical Translational Medicine of Ministry of Education, college of basic medical sciences, Hainan Medical University, Haikou 571199, China.

^†^ These authors contributed equally to this work.

^*^ Corresponding author: Peng Zhang, Department of Forensic Medicine & Key Laboratory of Tropical Translational Medicine of Ministry of Education, college of basic medical sciences, Hainan Medical University, Xueyuan Road 3#, Longhuaqu, Haikou, China. E-mail: 972421821@qq.com.

^*^ Corresponding author: Qianyun Nie, Department of Pathology & Key Laboratory of Tropical Translational Medicine of Ministry of Education, college of basic medical sciences, Hainan Medical University, Xueyuan Road 3#, Longhuaqu, Haikou, China. E-mail: nieqianyun0606@126.com.

1. **Figure S1.** Timeline of the experimental procedure and sample collection.
2. **Figure S2**. GO enrichment analysis bubble plot of differentially expressed proteins in the plasma of SD rats after acute SUDEP. The x-axis represents the enrichment degree, and the y-axis represents the GO term. The size of the circle is proportional to the number of differentially expressed proteins mapped to the corresponding GO term, and the color intensity reflects the P-value (the redder the color, the smaller the P-value).

3. **Table S1.** Identification of differentially expressed plasma proteins in the acute SUDEP group using dDIA combined with nanoLC-MS/MS technology.

4. **Table S2.** Stability of internal standard response in QC samples of SD rats with acute SUDEP.

5. **Table S3.** Differentially expressed lipids in the hippocampal tissue of chronic epilepsy group detected using UHPLC-QE-MS technology.


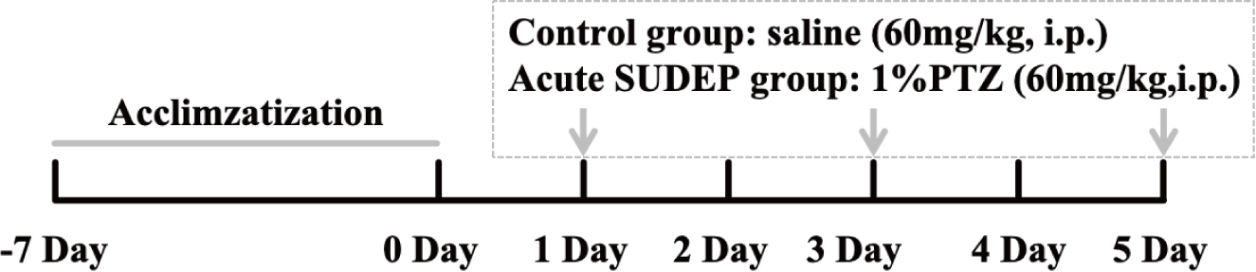


**Figure S1. Timeline of the experimental procedure and sample collection.**

**
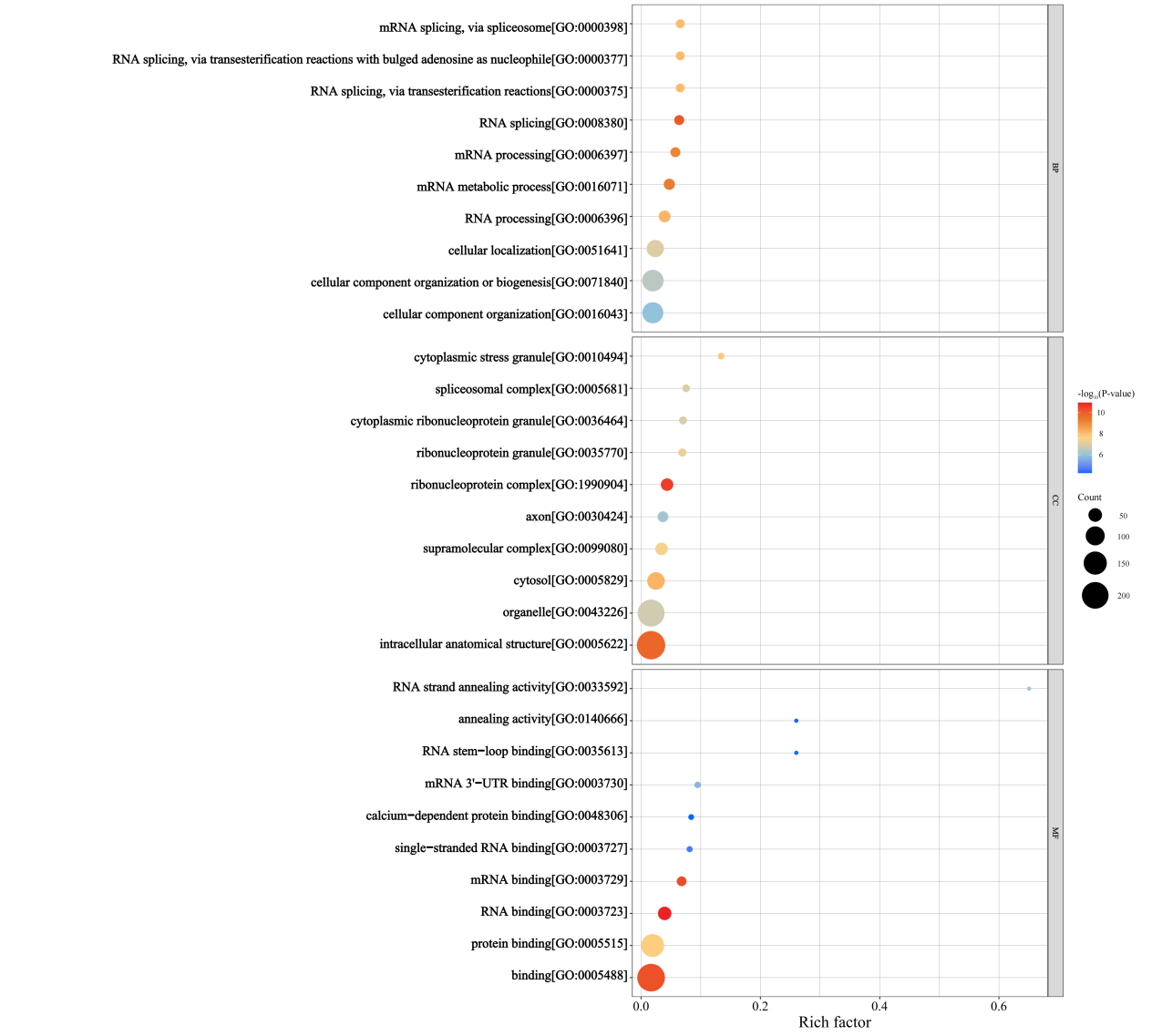
**

**Figure S1. GO enrichment analysis bubble plot of differentially expressed proteins in the plasma of SD rats after acute SUDEP.** The x-axis represents the enrichment degree, and the y-axis represents the GO term. The size of the circle is proportional to the number of differentially expressed proteins mapped to the corresponding GO term, and the color intensity reflects the P-value (the redder the color, the smaller the P-value).

Table S1. Identification of differentially expressed plasma proteins in the acute SUDEP group using dDIA combined with nanoLC-MS/MS technology.

| No. | Protein name | Gene name | Uniprot ID | Fold change | p-value |
| --- | --- | --- | --- | --- | --- |
| Up-regulated in the hippocampal tissue | | | | | |
| 1 | Phospholipase A2 (Fragment) | Pla2g2a | B6VNU1 | 9007.253399 | 0.004582786 |
| 2 | Alpha-fetoprotein | Afp | P02773 | 314.3241283 | 0.014305878 |
| 3 | Keratin, type II cytoskeletal 75 | Krt75 | Q6IG05 | 83.61505768 | 0.014305878 |
| 4 | E3 ubiquitin-protein ligase RNF8 | Rnf8 | Q4KLN8 | 83.39889601 | 0.014305878 |
| 5 | Heat shock 70kDa protein 4-like (Predicted), isoform CRA_b | Hspa4l | B4F772 | 76.04242782 | 0.014305878 |
| 6 | Photoreceptor cilium actin regulator | Pcare | D3ZS67 | 66.29171599 | 0.014305878 |
| 7 | Hepatic sodium/bile acid cotransporter | Slc10a1 | P26435 | 64.13570699 | 0.014305878 |
| 8 | Transient receptor potential cation channel subfamily A member 1 | Trpa1 | Q6RI86 | 61.78789328 | 0.014305878 |
| 9 | GC-rich sequence DNA-binding factor 2 | Gcfc2 | A0A8I6AHL1 | 52.28240444 | 0.014305878 |
| 10 | TIP41-like protein | Tiprl | A2VCX1 | 43.12062322 | 0.014305878 |
| 11 | Ketimine reductase mu-crystallin | Crym | Q9QYU4 | 32.67582311 | 0.014305878 |
| 12 | Interphotoreceptor matrix proteoglycan 1 | Impg1 | Q9ET62 | 30.17067773 | 0.014305878 |
| 13 | Ras-related protein Rab-3C | Rab3c | P62824 | 26.89049848 | 0.014305878 |
| 14 | Cysteine sulfinic acid decarboxylase | Csad | Q64611 | 9.048709855 | 0.01571884 |
| 15 | Guanine nucleotide-binding protein subunit beta-5 | Gnb5 | P62882 | 8.176422305 | 0.0477602 |
| 16 | Gamma-synuclein | Sncg | A0A0G2K0T6 | 7.44111344 | 0.004174105 |
| 17 | Ornithine aminotransferase, mitochondrial | Oat | P04182 | 5.792693203 | 0.00714075 |
| 18 | Dynamin-1 | Dnm1 | P21575 | 5.63242168 | 0.04618025 |
| 19 | C5a anaphylatoxin chemotactic receptor 1 | C5ar1 | P97520 | 5.623039541 | 0.014674838 |
| 20 | Sclerostin domain-containing protein 1 | Sostdc1 | Q642G2 | 5.576036236 | 0.014207226 |
| 21 | 1-phosphatidylinositol 4,5-bisphosphate phosphodiesterase | Plcb4 | A0A8I5Y961 | 5.499756804 | 0.008984396 |
| 22 | Phosphodiesterase | Pde6a | A6IXG6 | 5.042171711 | 0.033476189 |
| 23 | Src kinase-associated phosphoprotein 1 | Skap1 | Q4V7G1 | 4.823986233 | 0.013481271 |
| 24 | Medium-chain specific acyl-CoA dehydrogenase, mitochondrial | Acadm | P08503 | 3.636881904 | 0.045520243 |
| 25 | C-C motif chemokine ligand 24 | Ccl24 | A0A0G2JYH6 | 3.139564697 | 0.002025061 |

Continuation Table S1

| No. | Protein name | Gene name | Uniprot ID | Fold change | p-value |
| --- | --- | --- | --- | --- | --- |
| Up-regulated in the hippocampal tissue | | | | | |
| 26 | Ig-like domain-containing protein | ENSRNOG00000065156 | A0A8I5ZPC5 | 3.028260573 | 0.000570553 |
| 27 | Receptor expression-enhancing protein | Reep2 | A0A0G2K1L5 | 2.933933392 | 0.018914832 |
| 28 | Type II inositol 3,4-bisphosphate 4-phosphatase | Inpp4b | Q9QWG5 | 2.924621549 | 0.034714907 |
| 29 | Ifi47 protein | Ifi47 | Q6NYB8 | 2.619569757 | 0.036389031 |
| 30 | C-X-C motif chemokine ligand 12 | Cxcl12 | A0A8I6GDX4 | 2.524993123 | 0.016483701 |
| 31 | non-specific serine/threonine protein kinase | Nrk | F1LZB6 | 2.510275295 | 0.036015431 |
| 32 | RCG25681 | Clec3b | A6I4B3 | 2.494617277 | 0.0062508 |
| 33 | Biglycan | Bgn | P47853 | 2.443160089 | 0.022015971 |
| 34 | FYN-binding protein 1 | Fyb1 | D3ZIE4 | 2.432791423 | 0.008302983 |
| 35 | SPARC | Sparc | P16975 | 2.398588878 | 0.02359042 |
| 36 | Glutamine synthetase | Glul | P09606 | 2.369998079 | 0.049228526 |
| 37 | Src kinase-associated phosphoprotein 2 | Skap2 | Q920G0 | 2.260707635 | 0.014584773 |
| 38 | Voltage-gated potassium channel subunit beta-2 | Kcnab2 | P62483 | 2.233114073 | 0.0004798 |
| 39 | Hepatic triacylglycerol lipase | Lipc | P07867 | 2.20910548 | 0.038493946 |
| 40 | Adipocyte enhancer-binding protein 1 | Aebp1 | A2RUV9 | 2.197257282 | 0.018035254 |
| 41 | Signal peptide, CUB domain and EGF like domain containing 1 | Scube1 | A0A8I6A5S5 | 2.088873851 | 0.019761841 |
| 42 | non-specific serine/threonine protein kinase | Tnik | A0A8I6GLD8 | 2.037120639 | 0.015351111 |
| 43 | Formin-binding protein 1-like | Fnbp1l | A0A8L2QQ20 | 2.036753264 | 0.048338822 |
| 44 | Polymeric immunoglobulin receptor | Pigr | P15083 | 2.029432392 | 0.008665552 |
| 45 | Eukaryotic translation initiation factor 2 subunit 1 | Eif2s1 | P68101 | 1.970656368 | 0.00285433 |
| 46 | Formin-binding protein 1-like | Fnbp1l | Q2HWF0 | 1.963570467 | 0.032399337 |
| 47 | Ig-like domain-containing protein | ENSRNOG00000062976 | F1LYU3 | 1.951050359 | 0.000411965 |
| 48 | Phosphatidylcholine:ceramide cholinephosphotransferase 2 | Sgms2 | Q4JM44 | 1.92726692 | 0.011833835 |
| 49 | Extracellular sulfatase | Sulf2 | A0A8I6A3G4 | 1.912216236 | 0.047255241 |

Continuation Table S1

| No. | Protein name | Gene name | Uniprot ID | Fold change | p-value |
| --- | --- | --- | --- | --- | --- |
| Up-regulated in the hippocampal tissue | | | | | |
| 51 | Collagen type VI alpha 3 chain | Col6a3 | A0A8I5ZTR6 | 1.858704982 | 0.047352099 |
| 52 | ATP-citrate synthase | Acly | P16638 | 1.848653303 | 0.017056109 |
| 53 | Similar to RIKEN cDNA 1110020A23 | Rnasek | A0A8I5ZM61 | 1.8019928 | 0.045906857 |
| 54 | Serine/threonine-protein kinase TAO3 | Taok3 | Q53UA7 | 1.787458505 | 0.044815302 |
| 55 | Na(+)/H(+) exchange regulatory cofactor NHE-RF1 | Nherf1 | Q9JJ19 | 1.78716104 | 0.037076546 |
| 56 | 1-phosphatidylinositol 4,5-bisphosphate phosphodiesterase beta-4 | Plcb4 | Q9QW07 | 1.733211382 | 0.009374296 |
| 57 | Family with sequence similarity 114, member A2 | Fam114a2 | A0A8I6APK7 | 1.723332971 | 0.043938007 |
| 58 | Nuclear export mediator factor | Nemf | A0A8I6A2E1 | 1.71849017 | 0.040287892 |
| 59 | TBC1 domain family, member 10b (Predicted) | Tbc1d10b | A6I9M3 | 1.702010345 | 0.018208098 |
| 60 | Protein kinase C theta type | Prkcq | Q9WTQ0 | 1.613305087 | 0.011718921 |
| 61 | Tubulin alpha-8 chain | Tuba8 | Q6AY56 | 1.609919282 | 0.027021974 |
| 62 | Kinesin family member 21A | Kif21a | A0A8I6A1X3 | 1.604243843 | 0.020154678 |
| 63 | Arf-GAP with GTPase, ANK repeat and PH domain-containing protein 2 | Agap2 | Q8CGU4 | 1.548842508 | 0.021217926 |
| 64 | Proteasome subunit alpha type-4 | Psma4 | P21670 | 1.532760612 | 0.043159254 |
| 65 | Heterochromatin protein 1-binding protein 3 | Hp1bp3 | Q6P747 | 1.529737894 | 0.043484511 |
| Down-regulated in the hippocampal tissue | | | | | |
| 66 | Septin-9 | Septin9 | Q9QZR6 | 0.666294278 | 0.039421211 |
| 67 | AP-2 complex subunit alpha | Ap2a1 | A0A8I5ZVC3 | 0.665299093 | 0.02633745 |
| 68 | Protein CUSTOS | Custos | Q5I034 | 0.664733096 | 0.014037791 |
| 69 | Copper transport protein ATOX1 | Atox1 | Q9WUC4 | 0.663350797 | 0.029433609 |
| 70 | RCG48622, isoform CRA_a | Eml3 | A6HZX8 | 0.654612638 | 0.03623749 |
| 71 | Transcription factor BTF3 | Btf3 | A6I541 | 0.651371608 | 0.03002751 |
| 72 | IQ motif and SEC7 domain-containing protein 1 | Iqsec1 | A0A0G2JUG7 | 0.649958464 | 0.01504299 |
| 73 | AP2-associated protein kinase 1 | Aak1 | P0C1X8 | 0.649556085 | 0.044264189 |

Continuation Table S1

| No. | Protein name | Gene name | Uniprot ID | Fold change | p-value |
| --- | --- | --- | --- | --- | --- |
| Down-regulated in the hippocampal tissue | | | | | |
| 74 | WASH complex subunit 5 | Washc5 | F1M1B3 | 0.64853659 | 0.009929649 |
| 75 | Glutathione peroxidase 3 | Gpx3 | P23764 | 0.645095157 | 0.010541564 |
| 76 | Lysine--tRNA ligase | Kars1 | Q5XIM7 | 0.643060345 | 0.03034152 |
| 77 | Coatomer subunit delta | Arcn1 | Q66H80 | 0.640351832 | 0.027058607 |
| 78 | Fibrinogen, gamma polypeptide, isoform CRA_a | Fgg | A6J5U7 | 0.629212413 | 0.049755578 |
| 79 | Clathrin interactor 1 | Clint1 | A0A0G2JW94 | 0.621786169 | 0.049906445 |
| 80 | Ras-related protein Rab-31 | Rab31 | Q6GQP4 | 0.620402224 | 0.01814128 |
| 81 | C-terminal-binding protein 1 | Ctbp1 | Q9Z2F5 | 0.612892761 | 0.014974356 |
| 82 | Protein S100-A10 | S100a10 | P05943 | 0.608281282 | 0.02427414 |
| 83 | Cilia- and flagella-associated protein 20 | Cfap20 | A0A0H2UI05 | 0.607787776 | 0.0234233 |
| 84 | WASH complex subunit 1 | Washc1 | B2RYF7 | 0.607254271 | 0.003135501 |
| 85 | DnaJ homolog subfamily A member 2 | Dnaja2 | O35824 | 0.607112937 | 0.018226272 |
| 86 | Tight junction protein 2 | Tjp2 | A0A8I6ALD4 | 0.606567811 | 0.021980871 |
| 87 | Filamin B | Flnb | A0A0G2JXT8 | 0.604579456 | 0.04835288 |
| 88 | N-acetylneuraminate synthase | Nans | A0A8I6GKW3 | 0.598057693 | 0.01735515 |
| 89 | Acyl-protein thioesterase 1 | Lypla1 | P70470 | 0.591506389 | 0.049969895 |
| 90 | Septin | Septin7 | A2VCW8 | 0.588736812 | 0.046884893 |
| 91 | V-type proton ATPase subunit G | Atp6v1g1 | A0A8I6B3N3 | 0.588193698 | 0.020960127 |
| 92 | CAP-Gly domain-containing linker protein 1 | Clip1 | Q9JK25 | 0.588125095 | 0.016763034 |
| 93 | Mimecan | Ogn | A0A1W2Q6Q0 | 0.587833622 | 0.029579833 |
| 94 | Tight junction protein ZO-1 | Tjp1 | A0A0G2K2P5 | 0.579499716 | 0.009282557 |
| 95 | E2 ubiquitin-conjugating enzyme | Ube2e1 | A6K033 | 0.577976355 | 0.000355804 |
| 96 | DnaJ (Hsp40) homolog, subfamily B, member 4 | Dnajb4 | A0A9K3Y755 | 0.576510324 | 0.041937193 |
| 97 | RNA helicase | Ddx17 | A0A096MIX2 | 0.574802076 | 0.029122687 |
| 98 | SAM and SH3 domain containing 1 | Sash1 | A0A8I5Y723 | 0.573945668 | 0.015234988 |

Continuation Table S1

| No. | Protein name | Gene name | Uniprot ID | Fold change | p-value |
| --- | --- | --- | --- | --- | --- |
| Down-regulated in the hippocampal tissue | | | | | |
| 99 | Large ribosomal subunit protein eL24 | Rpl24 | P83732 | 0.571885147 | 0.028172663 |
| 100 | SWI/SNF related, matrix associated, actin dependent regulator of chromatin, subfamily a, member 2 | Smarca2 | A0A0G2JUS4 | 0.567162093 | 0.029934646 |
| 101 | Annexin A2 | Anxa2 | Q07936 | 0.566207069 | 0.041328584 |
| 102 | RNA-binding protein FXR1 | Fxr1 | Q5XI81 | 0.564459264 | 0.01319716 |
| 103 | protein-serine/threonine phosphatase | Ctdp1 | A0A0G2K0J7 | 0.560320344 | 0.030035026 |
| 104 | KN motif and ankyrin repeat domain-containing protein 2 | Kank2 | D3ZD05 | 0.559784106 | 0.004527833 |
| 105 | SNARE-associated protein Snapin | Snapin | P60192 | 0.551688396 | 0.046821241 |
| 106 | Protein BUD31 homolog | Bud31 | O70454 | 0.551478286 | 0.028621789 |
| 107 | RALY heterogeneous nuclear ribonucleoprotein | Raly | A0A0G2K974 | 0.549440599 | 0.00979368 |
| 108 | Mitochondrial import receptor subunit TOM34 | Tomm34 | Q3KRD5 | 0.548603999 | 0.015653301 |
| 109 | N-alpha-acetyltransferase 35, NatC auxiliary subunit | Naa35 | Q6DKG0 | 0.546982043 | 0.004907781 |
| 110 | Ig-like domain-containing protein | ENSRNOG00000067079 | A0A8I6AB22 | 0.546456715 | 0.010133892 |
| 111 | Transgelin | Tagln | P31232 | 0.545955063 | 0.014309199 |
| 112 | Importin subunit alpha-5 | Kpna1 | P83953 | 0.544586467 | 0.038544999 |
| 113 | Adenine phosphoribosyltransferase | Aprt | P36972 | 0.541396613 | 0.03084874 |
| 114 | DnaJ heat shock protein family (Hsp40) member C7 | Dnajc7 | A0A0G2K435 | 0.537085154 | 0.019862233 |
| 115 | Valine--tRNA ligase | Vars1 | Q04462 | 0.532389113 | 0.009258212 |
| 116 | DnaJ homolog subfamily C member 17 | Dnajc17 | D3ZSC8 | 0.532130793 | 0.043012618 |
| 117 | Carboxypeptidase B2 | Cpb2 | Q9EQV9 | 0.5311213 | 0.042644248 |
| 118 | Catenin alpha-1 | Ctnna1 | F7F7X1 | 0.53080199 | 0.012483162 |
| 119 | Sorting nexin-1 | Snx1 | Q99N27 | 0.528948276 | 0.040766023 |
| 120 | Xaa-Pro aminopeptidase 2 | Xpnpep2 | Q99MA2 | 0.527713173 | 0.021260907 |
| 121 | Peptidyl-prolyl cis-trans isomerase | Pin4 | A0A8I6GKI8 | 0.527415061 | 0.017307216 |
| 122 | Small ribosomal subunit protein eS17 | Rps17 | P04644 | 0.52700121 | 0.040761703 |
| 123 | WW domain-binding protein 4 | Wbp4 | Q5HZF2 | 0.526074464 | 0.017699128 |
| 124 | RNA-binding protein Luc7-like | Luc7l2 | B2RYP6 | 0.524221574 | 0.004820495 |

Continuation Table S1

| No. | Protein name | Gene name | Uniprot ID | Fold change | p-value |
| --- | --- | --- | --- | --- | --- |
| Down-regulated in the hippocampal tissue | | | | | |
| 125 | Large subunit GTPase 1 homolog | Lsg1 | Q5BJT6 | 0.522805587 | 0.032427778 |
| 126 | RCG51654, isoform CRA_a | Lsm6 | A6IYJ3 | 0.521573108 | 0.031470175 |
| 127 | RCG47974, isoform CRA_e | Dock1 | A6HX51 | 0.517842558 | 0.018859024 |
| 128 | RNA helicase | Ddx3x | A0A0G2K719 | 0.515635832 | 0.032922118 |
| 129 | Elongation factor 1-alpha 1 | Eef1a1 | P62630 | 0.514358203 | 0.000634468 |
| 130 | Tripartite motif-containing 25 | Trim25 | D4A9N5 | 0.510543668 | 0.033953978 |
| 131 | Receptor-type tyrosine-protein phosphatase alpha | Ptpra | Q03348 | 0.510009309 | 0.021383309 |
| 132 | TBC1 domain family, member 10a | Tbc1d10a | F7EVS3 | 0.509594847 | 0.000800906 |
| 133 | Small RNA binding exonuclease protection factor La | Ssb | A0A8I5ZZL0 | 0.504302914 | 0.020703623 |
| 134 | Annexin A5 | Anxa5 | P14668 | 0.497172485 | 0.010422885 |
| 135 | 28 kDa heat- and acid-stable phosphoprotein | Pdap1 | Q62785 | 0.490804931 | 0.021146133 |
| 136 | Coiled-coil domain-containing protein 93 | Ccdc93 | Q5BJT7 | 0.478997481 | 0.047708627 |
| 137 | Heterogeneous nuclear ribonucleoprotein H3 | Hnrnph3 | A0A0G2JVA2 | 0.478467769 | 0.046855785 |
| 138 | ATP-dependent RNA helicase DHX30 | Dhx30 | Q5BJS0 | 0.476720178 | 0.017729365 |
| 139 | Prolargin | Prelp | Q9EQP5 | 0.474780629 | 0.025737341 |
| 140 | Fragile X messenger ribonucleoprotein 1 | Fmr1 | Q80WE1 | 0.465999994 | 0.002932385 |
| 141 | Elongation factor 1-alpha 2 | Eef1a2 | P62632 | 0.46573621 | 0.000404744 |
| 142 | LUC7-like | Luc7l | A0A8I6A458 | 0.46427736 | 0.008460306 |
| 143 | HIV TAT specific factor 1 (Predicted) | Htatsf1 | A6KSQ2 | 0.463469026 | 0.024031408 |
| 144 | Adenylate kinase isoenzyme 1 | Ak1 | P39069 | 0.461948989 | 0.023403125 |
| 145 | Cdc42-interacting protein 4 | Trip10 | P97531 | 0.457908263 | 0.010871341 |
| 146 | Multiple inositol polyphosphate phosphatase 1 | Minpp1 | O35217 | 0.450850188 | 0.009288229 |
| 147 | Heterogeneous nuclear ribonucleoprotein L-like | Hnrnpll | D4A3E1 | 0.447831603 | 0.033277392 |
| 148 | Small ribosomal subunit protein uS10 | Rps20 | P60868 | 0.444696352 | 0.010633325 |
| 149 | Eukaryotic translation initiation factor 4H | Eif4h | Q5XI72 | 0.443790465 | 0.049832721 |
| 150 | AHNAK nucleoprotein | Ahnak | A0A0G2JUA5 | 0.443082133 | 0.034776918 |

Continuation Table S1

| No. | Protein name | Gene name | Uniprot ID | Fold change | p-value |
| --- | --- | --- | --- | --- | --- |
| Down-regulated in the hippocampal tissue | | | | | |
| 151 | Large ribosomal subunit protein eL38 | Rpl38 | P63174 | 0.441931593 | 0.043250947 |
| 152 | BRCA2 and CDKN1A-interacting protein | Bccip | A6HX34 | 0.44026848 | 0.03057642 |
| 153 | Zinc finger protein 183 | Rnf113a2 | A0A8I5ZN54 | 0.436576163 | 0.012041213 |
| 154 | Choline-phosphate cytidylyltransferase A | Pcyt1a | P19836 | 0.431610978 | 0.00023757 |
| 155 | Heterogeneous nuclear ribonucleoproteins C1/C2-like | Hnrnpc | A0A0G2JXW4 | 0.431367389 | 0.044592861 |
| 156 | Large ribosomal subunit protein uL14 | Rpl23 | P62832 | 0.428230197 | 0.014936984 |
| 157 | Protein-lysine 6-oxidase | Lox | P16636 | 0.428151829 | 0.042672488 |
| 158 | RCG39872, isoform CRA_e | rCG_39872 | A6I9X7 | 0.426419225 | 0.033916615 |
| 159 | Tripeptidyl-peptidase 2 | Tpp2 | Q64560 | 0.425770649 | 0.018860913 |
| 160 | RCG64255 (Fragment) | rCG_64255 | A6KF54 | 0.425759414 | 0.045020992 |
| 161 | Fatty acid-binding protein, adipocyte | Fabp4 | P70623 | 0.423581898 | 0.020236548 |
| 162 | FAM192A/Fyv6 N-terminal domain-containing protein | RGD1307433 | A6JY58 | 0.419993023 | 0.01575969 |
| 163 | Tubulin polymerization-promoting protein family member 3 | Tppp3 | Q5PPN5 | 0.419121249 | 0.013328909 |
| 164 | Biogenesis of lysosome-related organelles complex 1 subunit 3 | Bloc1s3 | D4A3V6 | 0.418052495 | 0.013674782 |
| 165 | Small ribosomal subunit protein uS17 | Rps11 | P62282 | 0.417031945 | 0.037478897 |
| 166 | Similar to XAP-5 protein (Predicted), isoform CRA_a | Fam50a | A6KRQ9 | 0.408188047 | 0.028368141 |
| 167 | Glutathione S-transferase Mu 5 | Gstm5 | Q9Z1B2 | 0.408161447 | 0.018207591 |
| 168 | WAS/WASL-interacting protein family member 1 | Wipf1 | Q6IN36 | 0.404289897 | 0.01952379 |
| 169 | Myosin light chain 1/3, skeletal muscle isoform | Myl1 | P02600 | 0.403841437 | 0.004014412 |
| 170 | PHD finger protein 23 | Phf23 | A0A8I5ZRG2 | 0.40179485 | 0.013581986 |
| 171 | Mitogen-activated protein kinase 3 | Mapk3 | P21708 | 0.39912555 | 0.018856373 |
| 172 | AHNAK nucleoprotein | Ahnak | A0A0G2JU96 | 0.399124222 | 0.040196216 |
| 173 | threonine--tRNA ligase | Tars3 | A0A8I6AAW1 | 0.399068879 | 0.039677261 |
| 174 | Lupus La protein homolog | Ssb | P38656 | 0.391996757 | 9.98064E-06 |

Continuation Table S1

| No. | Protein name | Gene name | Uniprot ID | Fold change | p-value |
| --- | --- | --- | --- | --- | --- |
| Down-regulated in the hippocampal tissue | | | | | |
| 175 | Pre-mRNA 3'-end-processing factor FIP1 | Fip1l1 | Q5U317 | 0.389147125 | 0.02612731 |
| 176 | Poly(A) binding protein, nuclear 1 | Pabpn1 | A0A8I6A5F1 | 0.38027793 | 0.001512143 |
| 177 | Heterogeneous nuclear ribonucleoprotein K | Hnrnpk | P61980 | 0.37783293 | 0.017288809 |
| 178 | Serine and arginine rich splicing factor 3 | Srsf3 | A0A0U1RRV7 | 0.377175547 | 0.026666609 |
| 179 | Similar to Ext1 | Ext1 | A6HRF2 | 0.373841036 | 0.001499285 |
| 180 | family interacting protein 5 | Rab11fip5 | A0A0G2K1W1 | 0.373303516 | 0.003373347 |
| 181 | Tankyrase 1 binding protein 1 | Tnks1bp1 | D3ZF26 | 0.371962522 | 0.028150025 |
| 182 | Heterogeneous nuclear ribonucleoprotein D-like | Hnrnpdl | Q3SWU3 | 0.368984354 | 0.036157079 |
| 183 | Family with sequence similarity 98, member B, pseudogene 1 | Fam98b-ps1 | A0A8I5ZPB4 | 0.366431109 | 0.013308237 |
| 184 | Large ribosomal subunit protein eL28 | Rpl28 | P17702 | 0.366140109 | 0.049235264 |
| 185 | Amidophosphoribosyltransferase | Ppat | P35433 | 0.359275546 | 0.038680574 |
| 186 | U4/U6.U5 small nuclear ribonucleoprotein 27 kDa protein | Snrnp27 | A0A8I6ADI8 | 0.353358475 | 0.022110339 |
| 187 | Similar to RIKEN cDNA 0610038D11 (Predicted), isoform CRA_a | RGD1309710_predicted | A6HZJ3 | 0.353200765 | 0.024279962 |
| 188 | Transcriptional regulator protein Pur-beta | Purb | Q68A21 | 0.353064346 | 0.025023688 |
| 189 | Peflin | Pef1 | Q641Z8 | 0.351339384 | 0.02987959 |
| 190 | Heterogeneous nuclear ribonucleoprotein M | Hnrnpm | A0A8I5ZQ38 | 0.348043615 | 0.041332064 |
| 191 | cAMP-regulated phosphoprotein 19 | Arpp19 | Q712U5 | 0.346493709 | 0.014305878 |
| 192 | RCG33491, isoform CRA_a | Zfp207 | A6HHB3 | 0.345834667 | 0.014305878 |
| 193 | Myosin-10 | Myh10 | Q9JLT0 | 0.343333818 | 0.014305878 |
| 194 | Endoglin | Eng | A0A8I6A813 | 0.342398426 | 0.014305878 |
| 195 | Neuropilin-2 | Nrp2 | O35276 | 0.341907939 | 0.014305878 |
| 196 | Annexin A3 | Anxa3 | P14669 | 0.340276296 | 0.014305878 |
| 197 | peptidylprolyl isomerase | Fkbp15 | A0A8I5ZXY2 | 0.338519362 | 0.018043651 |
| 198 | Carboxypeptidase N catalytic chain | Cpn1 | Q9EQV8 | 0.337831856 | 0.00503122 |

Continuation Table S1

| No. | Protein name | Gene name | Uniprot ID | Fold change | p-value |
| --- | --- | --- | --- | --- | --- |
| Down-regulated in the hippocampal tissue | | | | | |
| 199 | Glutathione S-transferase Mu 2 | Gstm2 | P08010 | 0.337541499 | 0.015704338 |
| 200 | General transcription factor II-I | Gtf2i | Q5U2Y1 | 0.335564836 | 0.034323457 |
| 201 | TATA-box binding protein associated factor 15 | Taf15 | A0A0G2K3Z7 | 0.335348206 | 0.026392946 |
| 202 | PTB domain-containing engulfment adapter protein 1 | Gulp1 | Q5PQS4 | 0.327709662 | 0.010153993 |
| 203 | Poly(A) binding protein, cytoplasmic 4 | Pabpc4 | A0A8I6ACF6 | 0.326511125 | 0.019926728 |
| 204 | Small ribosomal subunit protein eS10 | Rps10 | P63326 | 0.322633299 | 0.045872731 |
| 205 | Probable ATP-dependent RNA helicase DDX5 | Ddx5 | A0A8I5ZKV9 | 0.321329235 | 0.014305878 |
| 206 | POP1 homolog, ribonuclease P/MRP subunit | Pop1 | A0A0G2K4R5 | 0.320682139 | 0.024828546 |
| 207 | Coiled-coil domain-containing protein 25 | Ccdc25 | A6K6K7 | 0.313682357 | 0.014347008 |
| 208 | Unconventional myosin-Id | Myo1d | Q63357 | 0.308661347 | 0.044905284 |
| 209 | Splicing factor proline and glutamine rich | Sfpq | Q4KM71 | 0.308201807 | 0.03516455 |
| 210 | Ras association (RalGDS/AF-6) and pleckstrin homology domains 1 | Raph1 | A0A8I5ZL54 | 0.306789012 | 0.00101552 |
| 211 | Mitotic checkpoint protein BUB3 | Bub3 | A0A0G2JU63 | 0.304453076 | 0.014305878 |
| 212 | Cellular retinoic acid-binding protein 1 | Crabp1 | P62966 | 0.303266737 | 0.014305878 |
| 213 | Phosphoethanolamine/phosphocholine phosphatase 1 | Phospho1 | A0A8I6A6B0 | 0.293073063 | 0.014305878 |
| 214 | Doublecortin-like kinase 1 | Dclk1 | A0A0G2KB92 | 0.286870425 | 0.014305878 |
| 215 | Vigilin | Hdlbp | Q9Z1A6 | 0.281499296 | 0.014305878 |
| 216 | Microtubule-associated protein 4 | Map4 | Q5M7W5 | 0.277515286 | 0.017102382 |
| 217 | Dedicator of cytokinesis 5 | Dock5 | A0A8I5ZK89 | 0.274678093 | 0.014305878 |
| 218 | non-specific serine/threonine protein kinase | Nek9 | A0A8I6AQL7 | 0.273319148 | 0.047509808 |
| 219 | LSM14A mRNA processing body assembly factor | Lsm14a | A0A096MJY7 | 0.269763655 | 0.014305878 |

Table S2. Stability of internal standard response in QC samples of SD rats with acute SUDEP.

| Peaks No. | Retention time (s) | m/z | RSD (%) |
| --- | --- | --- | --- |
| IS1 | 96.2 | 183.0826 | 0.24 |
| IS2 | 158.8 | 133.1064 | 0.9 |
| IS3 | 227.7 | 152.06 | 0.51 |
| IS4 | 27.2 | 127.0804 | 0.4 |
| IS5 | 158.8 | 135.1207 | 1.24 |
| IS6 | 195 | 85.1322 | 0.57 |

Table S3. Differentially expressed lipids in the hippocampal tissue of chronic epilepsy group detected using UHPLC-QE-MS technology.

| No. | Metabolite | Retention time (s) | Ion (m/z) | VIP | P-value | Fold change | ESI mode |
| --- | --- | --- | --- | --- | --- | --- | --- |
| 1 | Isouron | 212.139 | 126.7 | 2.1 | 0.006 | 160.962 | ESI+ |
| 2 | PIP(16:2(9Z,12Z)/18:0) | 915.504 | 296.5 | 1.5 | 0.023 | 70.547 | ESI+ |
| 3 | Fraxetin | 207.029 | 197.5 | 2.1 | 0.003 | 54.100 | ESI- |
| 4 | Tyr-Phe | 327.130 | 168.2 | 2.1 | 0.007 | 52.963 | ESI- |
| 5 | Plumbagin | 187.042 | 14.4 | 1.8 | 0.009 | 26.320 | ESI- |
| 6 | Adrenochrome | 180.065 | 39.3 | 1.9 | 0.044 | 21.669 | ESI+ |
| 7 | 4-(4-Nitrophenyl)-1H-imidazole | 188.046 | 13.8 | 2.0 | 0.007 | 17.720 | ESI- |
| 8 | Fosinopril | 563.315 | 238.2 | 1.8 | 0.022 | 17.670 | ESI+ |
| 9 | 18.beta.-Glycyrrhetic acid methyl ester | 485.359 | 20.4 | 1.2 | 0.046 | 16.569 | ESI+ |
| 10 | Tsugaric_acid_B | 529.384 | 21.3 | 1.5 | 0.040 | 15.868 | ESI+ |
| 11 | Ile-Thr | 233.150 | 137.5 | 1.8 | 0.028 | 13.773 | ESI+ |
| 12 | Thr-Leu | 231.135 | 136.5 | 1.8 | 0.028 | 13.641 | ESI- |
| 13 | Met-Ala | 221.096 | 141.5 | 2.0 | 0.043 | 13.634 | ESI+ |
| 14 | Gln-Leu | 260.161 | 173.4 | 2.0 | 0.008 | 13.267 | ESI+ |
| 15 | Oxoundecanoylcarnitine (Car(11:1-O)) | 344.243 | 25.1 | 2.0 | 0.003 | 11.154 | ESI+ |
| 16 | Gly-Arg | 232.141 | 260.4 | 1.8 | 0.015 | 10.668 | ESI+ |
| 17 | LPI(20:4) | 621.304 | 147.3 | 2.1 | 0.002 | 9.254 | ESI+ |
| 18 | Maltoxazine | 180.102 | 141.4 | 2.1 | 0.000 | 9.207 | ESI+ |
| 19 | N,N-Dimethylsphingosine | 328.321 | 29.3 | 2.2 | 0.000 | 8.697 | ESI+ |
| 20 | Metanephrine | 198.113 | 141.6 | 2.2 | 0.000 | 7.593 | ESI+ |
| 21 | N-Lactoyl-Phenylalanine | 236.093 | 72.4 | 2.0 | 0.004 | 7.574 | ESI- |
| 22 | N-Lactoylphenylalanine | 236.093 | 72.4 | 2.0 | 0.004 | 7.574 | ESI- |
| 23 | Glycochenodeoxycholate 7-sulfate | 547.307 | 146.2 | 1.9 | 0.001 | 7.106 | ESI+ |
| 24 | 6a,9-Dihydroxy-2-(3-hydroxyprop-1-en-2-yl)-8-methoxy-1,2,12,12a-tetrahydrochromeno[3,4-b]furo[2,3-h]chromen-6(6aH)-one | 411.110 | 188.8 | 1.7 | 0.026 | 6.968 | ESI- |
| 25 | 2'-Deoxyguanosine 5'-monophosphate (dGMP) | 346.056 | 251.9 | 2.1 | 0.002 | 6.786 | ESI- |

Continuation Table S3

| No. | Metabolite | Retention time (s) | Ion (m/z) | VIP | P-value | Fold change | ESI mode |
| --- | --- | --- | --- | --- | --- | --- | --- |
| 26 | 6,6'-Dihydroxy-5,5'-dimethoxybiphenyl-3,3'-dicarboxylic acid | 333.064 | 15.6 | 1.5 | 0.013 | 6.756 | ESI- |
| 27 | Val-Thr | 219.134 | 152.7 | 1.8 | 0.013 | 6.533 | ESI+ |
| 28 | 1-Palmitoyl-2-hydroxy-sn-glycero-3-phospho-(1'-rac-glycerol) | 483.274 | 46.5 | 2.0 | 0.001 | 6.330 | ESI- |
| 29 | Corymbiferin | 303.053 | 15.1 | 1.6 | 0.006 | 6.291 | ESI- |
| 30 | 1H-Naphtho[2,3-c]pyran-3-acetic acid, 3,4,4a,5,10,10a-hexahydro-4a,9,10a-trihydroxy-1-methyl-5,10-dioxo- | 335.079 | 20.9 | 1.2 | 0.008 | 6.217 | ESI- |
| 31 | 2-((3aR,4S,7R,7aS)-1,3-Dioxohexahydro-1H-4,7-methanoisoindol-2(3H)-yl)propanoic acid | 236.093 | 85.7 | 2.0 | 0.002 | 6.198 | ESI- |
| 32 | Salannin | 597.304 | 149.5 | 1.8 | 0.001 | 6.102 | ESI- |
| 33 | Val-Ile | 229.156 | 104.8 | 1.8 | 0.036 | 6.023 | ESI- |
| 34 | Leu-Val | 229.156 | 104.8 | 1.8 | 0.036 | 6.023 | ESI- |
| 35 | Thiolutin | 226.997 | 57.1 | 1.4 | 0.005 | 5.977 | ESI- |
| 36 | D-myo-Inositol, 1-[2-hydroxy-3-[(1-oxo-9,12-octadecadienyl)oxy]propyl hydrogen phosphate], [S-(Z,Z)]- | 595.291 | 154.5 | 2.0 | 0.004 | 5.919 | ESI- |
| 37 | 2,2'-(1H-1,2,4-Triazole-3,5-diyl)diphenol | 252.074 | 87.7 | 1.7 | 0.000 | 5.839 | ESI- |
| 38 | 1,3,7-Trimethyluric acid | 209.068 | 87.9 | 1.7 | 0.000 | 5.824 | ESI- |
| 39 | Dehydroascorbic acid (Oxidized vitamin C) | 173.009 | 55.2 | 1.5 | 0.011 | 5.771 | ESI- |
| 40 | Sph(d18:0) | 302.305 | 51.2 | 2.1 | 0.001 | 5.734 | ESI+ |
| 41 | Adenosine monophosphate (AMP) | 348.071 | 252.2 | 2.1 | 0.001 | 5.722 | ESI+ |
| 42 | Adenosine 3'-monophosphate | 348.071 | 252.2 | 2.1 | 0.001 | 5.722 | ESI+ |
| 43 | 2,2',6,6'-Tetra-tert-butyldiphenylquinone | 409.307 | 45.2 | 1.8 | 0.002 | 5.647 | ESI+ |

Continuation Table S3

| No. | Metabolite | Retention time (s) | Ion (m/z) | VIP | P-value | Fold change | ESI mode |
| --- | --- | --- | --- | --- | --- | --- | --- |
| 44 | N6-(DELTA2-ISOPENTENYL)-ADENINE | 202.109 | 65.1 | 1.5 | 0.025 | 5.589 | ESI- |
| 45 | 2,2'-[(Phosphonomethyl)imino]diacetic acid | 226.013 | 57.1 | 1.5 | 0.006 | 5.499 | ESI- |
| 46 | Topiramate | 338.093 | 167.7 | 2.2 | 0.000 | 5.298 | ESI- |
| 47 | PS(20:1(11Z)/14:1(9Z)) | 760.513 | 134.6 | 2.0 | 0.002 | 5.260 | ESI+ |
| 48 | Sph(t18:0) | 318.300 | 53.9 | 2.2 | 0.000 | 4.992 | ESI+ |
| 49 | Dihydroorotic acid | 157.026 | 167.7 | 2.2 | 0.000 | 4.953 | ESI- |
| 50 | DEOXYRIBOSE | 133.051 | 92.8 | 1.9 | 0.007 | 4.952 | ESI- |
| 51 | Sphingosine | 300.290 | 30.6 | 2.1 | 0.001 | 4.931 | ESI+ |
| 52 | Dodemorph | 282.279 | 30.7 | 2.0 | 0.001 | 4.929 | ESI+ |
| 53 | 4-O-p-Coumaroylquinic acid | 337.090 | 167.7 | 2.2 | 0.000 | 4.731 | ESI- |
| 54 | M227T102 | 226.997 | 102.1 | 1.6 | 0.004 | 4.574 | ESI- |
| 55 | 1-Stearoyl-2-arachidonoyl-sn-glycero-3-phosphoserine | 810.530 | 113.4 | 2.1 | 0.000 | 4.565 | ESI- |
| 56 | Cysteine S-sulfate | 199.970 | 179.7 | 1.7 | 0.015 | 4.496 | ESI- |
| 57 | 1H-Indole-4-carboxamide, N-[(1,2-dihydro-4,6-dimethyl-2-oxo-3-pyridinyl)methyl]-3-methyl-1-[(1S)-1-methylpropyl]-6-[6-(1-piperazinyl)-3-pyridinyl]- | 525.305 | 151.7 | 2.1 | 0.001 | 4.430 | ESI- |
| 58 | Phe-Met-Arg-Phe-amide | 597.306 | 153.2 | 2.1 | 0.000 | 4.367 | ESI- |
| 59 | Ala-Glu | 217.083 | 177.1 | 1.8 | 0.002 | 4.237 | ESI- |
| 60 | Dihydrojasmonic Acid | 230.175 | 48.5 | 1.2 | 0.044 | 4.162 | ESI+ |
| 61 | 4-(Methylnitrosamino)-1-(3-pyridyl)-1-butanol | 210.124 | 150.8 | 1.8 | 0.011 | 4.126 | ESI+ |
| 62 | 5-Chloro-7-(4-morpholinylmethyl)-8-quinolinol | 277.074 | 142.8 | 2.2 | 0.000 | 4.080 | ESI- |
| 63 | 21-Deoxycortisol | 405.229 | 19 | 2.0 | 0.001 | 4.049 | ESI- |
| 64 | Sph(d18:2) | 298.274 | 31 | 1.7 | 0.005 | 4.016 | ESI+ |
| 65 | N-(1H-Tetraazol-5-yl)decanamide | 240.181 | 26 | 2.1 | 0.000 | 4.008 | ESI+ |
| 66 | 2-Dimethylamino-6-hydroxypurine | 180.087 | 48.7 | 2.0 | 0.004 | 3.977 | ESI+ |

Continuation Table S3

| No. | Metabolite | Retention time (s) | Ion (m/z) | VIP | P-value | Fold change | ESI mode |
| --- | --- | --- | --- | --- | --- | --- | --- |
| 67 | N-Methyl-N-[2-oxo-2-(1-pyrrolidinyl)ethyl]amine | 143.118 | 182.8 | 1.6 | 0.006 | 3.833 | ESI+ |
| 68 | Linoleamide | 280.264 | 31.2 | 1.9 | 0.005 | 3.778 | ESI+ |
| 69 | 4-Chloro-N-(2-methoxy-5-methylphenyl)benzenesulfonamide | 310.033 | 88.3 | 1.6 | 0.001 | 3.777 | ESI- |
| 70 | 6-Tuliposide B | 310.115 | 143.6 | 2.0 | 0.003 | 3.752 | ESI- |
| 71 | Leucyl-Hydroxyproline | 245.150 | 182.1 | 1.9 | 0.001 | 3.733 | ESI+ |
| 72 | 3-{3-oxo-2H,3H-[1,2,4]Triazolo[4,3-a]pyridin-2-yl}propanoic acid | 208.072 | 85.1 | 2.0 | 0.004 | 3.723 | ESI+ |
| 73 | Acadesine (Drug) | 259.104 | 100.6 | 1.6 | 0.005 | 3.717 | ESI+ |
| 74 | Convallatoxin | 551.287 | 144.7 | 1.6 | 0.013 | 3.696 | ESI+ |
| 75 | M537T133 | 537.338 | 133 | 1.9 | 0.000 | 3.694 | ESI+ |
| 76 | Nigrifortine | 509.288 | 45.9 | 2.1 | 0.000 | 3.691 | ESI+ |
| 77 | Heptadecasphing-4-enine | 286.274 | 30.7 | 1.8 | 0.009 | 3.654 | ESI+ |
| 78 | 5-(4-Chlorobenzylidene)-2,4,6(1H,3H,5H)-pyrimidinetrione | 249.008 | 16.4 | 1.6 | 0.036 | 3.633 | ESI- |
| 79 | 5-Nitro-N-(3-pyridinylmethyl)-2-furamide | 248.064 | 88.9 | 1.9 | 0.006 | 3.516 | ESI+ |
| 80 | Met-Thr | 249.092 | 150.2 | 1.3 | 0.030 | 3.448 | ESI- |
| 81 | Ganoderic_acid_L | 535.321 | 134.6 | 2.2 | 0.000 | 3.419 | ESI+ |
| 82 | (S)-5-Methylhydantoin | 113.036 | 167.7 | 2.2 | 0.000 | 3.408 | ESI- |
| 83 | Nonenoylcarnitine (Car(9:1)) | 300.217 | 125.3 | 1.3 | 0.019 | 3.353 | ESI+ |
| 84 | .DELTA.8-Tetrahydrocannabinol | 315.228 | 156.4 | 1.6 | 0.024 | 3.347 | ESI+ |
| 85 | R-Palmitoyl-(2-methyl) ethanolamide | 314.306 | 29.4 | 1.7 | 0.003 | 3.314 | ESI+ |
| 86 | 1-Acetylpiperidine-2-carboxylic acid | 172.097 | 135.2 | 2.0 | 0.000 | 3.254 | ESI+ |
| 87 | N-Oleoyl-D-erythro-sphingosylphosphorylcholine | 729.591 | 26.1 | 2.0 | 0.006 | 3.229 | ESI+ |
| 88 | [1,2,4]Triazolo[4,3-a]quinazolin-5(1H)-one, 2,4-dihydro-4-methyl-1-thioxo- | 231.034 | 13 | 1.9 | 0.003 | 3.220 | ESI- |

Continuation Table S3

| No. | Metabolite | Retention time (s) | Ion (m/z) | VIP | P-value | Fold change | ESI mode |
| --- | --- | --- | --- | --- | --- | --- | --- |
| 89 | 4-(.beta.-D-Glucopyranosyloxy)pentan-2-yl (2E)-3-(4-hydroxyphenyl)prop-2-enoate | 411.167 | 98.2 | 1.4 | 0.047 | 3.213 | ESI- |
| 90 | Gly-His | 211.083 | 195.7 | 1.6 | 0.019 | 3.210 | ESI- |
| 91 | [(4-Methylbenzyl)sulfanyl]acetic acid | 195.051 | 206 | 1.7 | 0.010 | 3.204 | ESI- |
| 92 | Jineol | 162.055 | 22 | 1.5 | 0.039 | 3.171 | ESI+ |
| 93 | 8-Hydroxycarbostyril | 162.055 | 22 | 1.5 | 0.039 | 3.171 | ESI+ |
| 94 | 5-Phenylisoxazol-3-ol | 162.055 | 22 | 1.5 | 0.039 | 3.171 | ESI+ |
| 95 | 4-hydroxy-1H-indole-3-carbaldehyde | 162.055 | 22 | 1.5 | 0.039 | 3.171 | ESI+ |
| 96 | 1,5-Isoquinolinediol | 162.055 | 22 | 1.5 | 0.039 | 3.171 | ESI+ |
| 97 | Gestoden | 311.205 | 125.3 | 1.6 | 0.010 | 3.137 | ESI+ |
| 98 | Altersetin | 400.248 | 48.5 | 1.7 | 0.007 | 3.134 | ESI+ |
| 99 | Lyso-sphingomyelin | 465.345 | 131 | 2.0 | 0.002 | 3.089 | ESI+ |
| 100 | 2,4-Dimethylphenol | 121.066 | 13.1 | 1.5 | 0.029 | 3.089 | ESI- |
| 101 | Nadolol | 310.201 | 125.3 | 1.6 | 0.010 | 3.075 | ESI+ |
| 102 | Thymine | 125.036 | 29.6 | 1.9 | 0.011 | 3.069 | ESI- |
| 103 | Furcelleran | 478.198 | 141.5 | 1.8 | 0.001 | 3.045 | ESI+ |
| 104 | Argininosuccinic acid | 291.130 | 264.1 | 1.7 | 0.027 | 3.044 | ESI+ |
| 105 | Lotaustralin | 262.129 | 230.6 | 1.9 | 0.001 | 3.017 | ESI+ |
| 106 | 2-(Anilinocarbothioyl)-N-phenylhydrazinecarboxamide | 285.083 | 190.4 | 1.5 | 0.020 | 2.998 | ESI- |
| 107 | Adenosine 3',5'-cyclic phosphate (cAMP) | 328.046 | 170.8 | 2.0 | 0.000 | 2.995 | ESI- |
| 108 | Methanethiol | 84.951 | 90.2 | 1.3 | 0.031 | 2.973 | ESI- |
| 109 | 1H-Pyrrolo[3,4-b]quinoline-1,9(4H)-dione, 2,3-dihydro-4-methyl-3-(2-methylpropyl)- | 271.140 | 191.6 | 1.5 | 0.026 | 2.963 | ESI+ |
| 110 | 1-Propanone, 1-[4-(5'-chloro-3,5-dimethyl[2,4'-bipyridin]-2'-yl)-1-piperazinyl]-3-(methylsulfonyl)- | 437.142 | 206 | 1.9 | 0.005 | 2.948 | ESI+ |
| 111 | Citrinin | 249.079 | 126.6 | 1.9 | 0.000 | 2.947 | ESI- |
| 112 | 4′-Hydroxy-2′-methylacetophenone | 149.061 | 33.8 | 1.4 | 0.046 | 2.927 | ESI- |

Continuation Table S3

| No. | Metabolite | Retention time (s) | Ion (m/z) | VIP | P-value | Fold change | ESI mode |
| --- | --- | --- | --- | --- | --- | --- | --- |
| 113 | 4'-Hydroxy-3'-methylacetophenone | 149.061 | 33.8 | 1.4 | 0.046 | 2.927 | ESI- |
| 114 | 4-Allylcatechol | 149.061 | 33.8 | 1.4 | 0.046 | 2.927 | ESI- |
| 115 | 3-Phenylpropanoic acid | 149.061 | 33.8 | 1.4 | 0.046 | 2.927 | ESI- |
| 116 | 2'-Hydroxy-5'-methylacetophenone | 149.061 | 33.8 | 1.4 | 0.046 | 2.927 | ESI- |
| 117 | 2'-Hydroxy-4'-methylacetophenone | 149.061 | 33.8 | 1.4 | 0.046 | 2.927 | ESI- |
| 118 | 2,6-Dimethyl-4-hydroxybenzaldehyde | 149.061 | 33.8 | 1.4 | 0.046 | 2.927 | ESI- |
| 119 | Leu-Ile | 245.186 | 94.9 | 1.4 | 0.050 | 2.916 | ESI+ |
| 120 | Ile-Leu | 245.186 | 94.9 | 1.4 | 0.050 | 2.916 | ESI+ |
| 121 | 2-Aminobutyric acid | 104.071 | 193.1 | 1.4 | 0.024 | 2.903 | ESI+ |
| 122 | (4S,5Z,6S)-4-(2-methoxy-2-oxoethyl)-5-[2-[(E)-3-phenylprop-2-enoyl]oxyethylidene]-6-[(2S,3R,4S,5S,6R)-3,4,5-trihydroxy-6-(hydroxymethyl)oxan-2-yl]oxy-4H-pyran-3-carboxylic acid | 215.033 | 143.2 | 1.9 | 0.002 | 2.889 | ESI- |
| 123 | LPC(20:4) | 544.340 | 39.2 | 1.2 | 0.028 | 2.878 | ESI+ |
| 124 | 2-Naphthalenecarboxamide, N-[2-(4-oxo-1-phenyl-1,3,8-triazaspiro[4.5]dec-8-yl)ethyl]- | 427.219 | 158.1 | 1.5 | 0.027 | 2.865 | ESI- |
| 125 | Heptadecenoylcarnitine (Car(17:1)) | 412.342 | 92.6 | 2.0 | 0.002 | 2.844 | ESI+ |
| 126 | 2-Methylpropanamine | 74.096 | 146.1 | 1.4 | 0.014 | 2.838 | ESI+ |
| 127 | Rubraflavone_B | 475.248 | 182.8 | 1.3 | 0.017 | 2.822 | ESI+ |
| 128 | M147T173_1 | 147.030 | 172.5 | 1.5 | 0.031 | 2.809 | ESI- |
| 129 | Dehydroabietic acid | 301.212 | 159.9 | 1.7 | 0.016 | 2.807 | ESI+ |
| 130 | CROTONIC ACID | 85.030 | 153.9 | 1.9 | 0.004 | 2.788 | ESI- |
| 131 | 1,2,3,4-Tetrakis(4-pyridyl)thiophene | 393.116 | 206 | 1.9 | 0.002 | 2.770 | ESI+ |
| 132 | 1-Ethyl-3-methylimidazolium cation | 111.092 | 18 | 1.9 | 0.000 | 2.744 | ESI+ |
| 133 | M455T214 | 455.064 | 213.5 | 1.6 | 0.005 | 2.717 | ESI- |
| 134 | Formylmethionine | 176.039 | 96.2 | 1.9 | 0.003 | 2.713 | ESI- |
| 135 | Sch 210972 | 446.260 | 158.4 | 1.6 | 0.040 | 2.703 | ESI+ |

Continuation Table S3

| No. | Metabolite | Retention time (s) | Ion (m/z) | VIP | P-value | Fold change | ESI mode |
| --- | --- | --- | --- | --- | --- | --- | --- |
| 136 | Quercetin-3,7,3',4'-tetramethyl ether | 357.102 | 34.9 | 1.9 | 0.002 | 2.701 | ESI- |
| 137 | 4-Butyloxazole | 126.091 | 182.8 | 1.4 | 0.014 | 2.696 | ESI+ |
| 138 | Mannitol | 181.072 | 168.1 | 1.7 | 0.003 | 2.668 | ESI- |
| 139 | Iditol | 181.072 | 168.1 | 1.7 | 0.003 | 2.668 | ESI- |
| 140 | Glucitol | 181.072 | 168.1 | 1.7 | 0.003 | 2.668 | ESI- |
| 141 | Galactitol | 181.072 | 168.1 | 1.7 | 0.003 | 2.668 | ESI- |
| 142 | Leonubiastrin | 415.180 | 95 | 1.4 | 0.022 | 2.639 | ESI- |
| 143 | Palmitoylcarnitine (Car(16:0)) | 400.342 | 94.4 | 2.1 | 0.000 | 2.631 | ESI+ |
| 144 | PS(18:1/18:1) | 786.528 | 57.4 | 1.5 | 0.016 | 2.613 | ESI- |
| 145 | Uridine | 245.077 | 65.5 | 1.4 | 0.025 | 2.610 | ESI+ |
| 146 | Nivalenol | 335.111 | 153.5 | 1.5 | 0.030 | 2.610 | ESI+ |
| 147 | M555T38 | 555.274 | 37.6 | 1.9 | 0.000 | 2.598 | ESI- |
| 148 | Dimethyltin oxide | 166.951 | 199.1 | 2.2 | 0.000 | 2.589 | ESI+ |
| 149 | Maleic hydrazide | 113.035 | 65.5 | 1.4 | 0.024 | 2.578 | ESI+ |
| 150 | 2-Oleoyl-1-stearoyl-sn-glycero-3-phosphoserine | 790.558 | 129.3 | 1.9 | 0.002 | 2.573 | ESI+ |
| 151 | Sepiapterin | 238.094 | 147.1 | 1.9 | 0.000 | 2.571 | ESI+ |
| 152 | Biopterin | 238.094 | 147.1 | 1.9 | 0.000 | 2.571 | ESI+ |
| 153 | 2-Bromo-4-methoxyaniline | 201.984 | 180.1 | 1.5 | 0.014 | 2.569 | ESI+ |
| 154 | Bisphenol A diglycidyl ether | 358.207 | 155.4 | 1.6 | 0.033 | 2.543 | ESI+ |
| 155 | M570T122 | 570.342 | 122 | 2.0 | 0.000 | 2.515 | ESI- |
| 156 | N(alpha)-gamma-L-Glutamylhistamine | 277.074 | 160.2 | 1.9 | 0.000 | 2.511 | ESI- |
| 157 | Amoxicillin | 364.092 | 175.5 | 2.0 | 0.000 | 2.505 | ESI- |
| 158 | 3-Dehydroquinic acid | 171.028 | 214.2 | 2.0 | 0.000 | 2.500 | ESI- |
| 159 | LPE(O-18:2) | 464.313 | 112.6 | 2.1 | 0.000 | 2.495 | ESI+ |
| 160 | Piperazine | 87.092 | 253.4 | 1.6 | 0.005 | 2.490 | ESI+ |
| 161 | (3beta,5alpha,6beta,22E,24R)-23-Methylergosta-7,22-diene-3,5,6-triol | 445.368 | 15.4 | 1.3 | 0.000 | 2.479 | ESI+ |
| 162 | PC(8:0/8:0) | 492.309 | 123.3 | 2.0 | 0.000 | 2.478 | ESI+ |
| 163 | 3,4-Thiophenedicarbonitrile | 135.003 | 214.3 | 2.1 | 0.000 | 2.471 | ESI+ |

Continuation Table S3

| No. | Metabolite | Retention time (s) | Ion (m/z) | VIP | P-value | Fold change | ESI mode |
| --- | --- | --- | --- | --- | --- | --- | --- |
| 164 | 2-Propyl-4-pentenoic acid | 141.092 | 27.7 | 1.9 | 0.001 | 2.470 | ESI- |
| 165 | Arachidonoylcarnitine (Car(20:4)) | 448.342 | 87.9 | 1.9 | 0.000 | 2.461 | ESI+ |
| 166 | M335T214 | 335.034 | 214.2 | 2.1 | 0.000 | 2.449 | ESI- |
| 167 | 5-Hydroxy-1-tetralone | 161.061 | 36.2 | 1.4 | 0.008 | 2.441 | ESI- |
| 168 | 1-Pentanone, 1-(3,4-dihydroxyphenyl)-2-(1-pyrrolidinyl)- | 262.145 | 33.9 | 1.3 | 0.042 | 2.440 | ESI- |
| 169 | 2,2'-Dihydroxy-4-methoxybenzophenone | 243.063 | 65.7 | 1.3 | 0.037 | 2.430 | ESI- |
| 170 | Gluconic acid | 195.051 | 220.9 | 1.5 | 0.014 | 2.429 | ESI- |
| 171 | PC(14:0/P-16:0) | 690.552 | 27.3 | 1.8 | 0.003 | 2.421 | ESI+ |
| 172 | (-)-Jasmonic_acid | 209.119 | 31.6 | 1.9 | 0.000 | 2.395 | ESI- |
| 173 | LPC(O-22:1) | 564.438 | 99.6 | 1.5 | 0.002 | 2.389 | ESI+ |
| 174 | PC(14:0/16:0) | 706.538 | 37.8 | 2.1 | 0.000 | 2.386 | ESI+ |
| 175 | PC(10:0/10:0) | 548.371 | 116.5 | 1.8 | 0.000 | 2.384 | ESI+ |
| 176 | tert-Butyl 4-amino-3-(trifluoromethyl)phenylcarbamate | 277.118 | 48.6 | 1.6 | 0.034 | 2.382 | ESI+ |
| 177 | (2-aminoethoxy)[3-[hexadec-1-en-1-yloxy]-2-[icosa-5.8.11.14-tetraenoyloxy]propoxy]phosphinic acid | 722.514 | 35.5 | 2.1 | 0.000 | 2.371 | ESI- |
| 178 | Xylitol | 151.061 | 126 | 1.6 | 0.004 | 2.367 | ESI- |
| 179 | Ribitol | 151.061 | 126 | 1.6 | 0.004 | 2.367 | ESI- |
| 180 | Arabitol | 151.061 | 126 | 1.6 | 0.004 | 2.367 | ESI- |
| 181 | Didemethylmifepristone | 402.238 | 183.5 | 1.5 | 0.011 | 2.357 | ESI+ |
| 182 | (E)-3-[2-[(2S,3R,4S,5S,6R)-3,4,5-trihydroxy-6-(hydroxymethyl)oxan-2-yl]oxyphenyl]prop-2-enoic acid | 325.090 | 137 | 1.4 | 0.042 | 2.347 | ESI- |
| 183 | LPC(12:0) | 440.277 | 128 | 1.6 | 0.019 | 2.341 | ESI+ |
| 184 | N-Acetylcarnosine | 269.125 | 194.5 | 1.8 | 0.005 | 2.339 | ESI+ |
| 185 | Dihydrokaempferol | 287.053 | 180.4 | 2.0 | 0.000 | 2.334 | ESI- |
| 186 | LPS(18:1) | 524.299 | 152.7 | 1.5 | 0.012 | 2.331 | ESI+ |
| 187 | M146T152 | 146.081 | 151.9 | 1.5 | 0.026 | 2.324 | ESI+ |
| 188 | Citraconic acid | 129.020 | 30.2 | 1.8 | 0.002 | 2.323 | ESI- |

Continuation Table S3

| No. | Metabolite | Retention time (s) | Ion (m/z) | VIP | P-value | Fold change | ESI mode |
| --- | --- | --- | --- | --- | --- | --- | --- |
| 189 | 1-(10Z-Heptadecenoyl)-sn-glycero-3-phospho-(1'-rac-glycerol) | 495.280 | 15.7 | 1.7 | 0.001 | 2.321 | ESI- |
| 190 | Nicotinamide | 123.055 | 27 | 2.0 | 0.002 | 2.320 | ESI+ |
| 191 | Aminofructose_6-phosphate | 260.053 | 142.6 | 1.8 | 0.004 | 2.312 | ESI+ |
| 192 | Linoleoylcarnitine | 424.342 | 92.2 | 2.1 | 0.000 | 2.306 | ESI+ |
| 193 | CPA(18:2(9Z,12Z)/0:0) | 417.240 | 166.6 | 1.6 | 0.014 | 2.303 | ESI+ |
| 194 | Normetanephrine | 166.086 | 135.2 | 1.8 | 0.001 | 2.301 | ESI+ |
| 195 | (22E,24R)-Stigmasta-4,22-diene-3,6-dione | 425.346 | 92.2 | 2.1 | 0.000 | 2.286 | ESI+ |
| 196 | Bilobalide A | 349.090 | 206.8 | 1.8 | 0.006 | 2.257 | ESI+ |
| 197 | Nandrolone | 275.197 | 160.4 | 1.6 | 0.019 | 2.256 | ESI+ |
| 198 | M417T215 | 417.037 | 214.8 | 2.1 | 0.000 | 2.255 | ESI- |
| 199 | Guanidoacetic acid | 118.061 | 209.6 | 2.0 | 0.000 | 2.250 | ESI+ |
| 200 | Methyl_(R)-8-Hydroxy-9-decene-4,6-diynoate_glucoside | 355.136 | 137.2 | 1.7 | 0.005 | 2.249 | ESI+ |
| 201 | AS_1-5 | 716.567 | 27 | 1.9 | 0.001 | 2.248 | ESI+ |
| 202 | 2-(4-(Methylamino)phenyl)benzo[d]thiazol-6-ol | 257.075 | 130 | 1.8 | 0.005 | 2.244 | ESI+ |
| 203 | LysoPE(20:4(8Z,11Z,14Z,17Z)/0:0) | 502.293 | 120.5 | 2.1 | 0.000 | 2.234 | ESI+ |
| 204 | M187T198 | 187.002 | 198.4 | 2.2 | 0.000 | 2.232 | ESI- |
| 205 | Isoxanthopterin | 180.049 | 167.4 | 1.7 | 0.005 | 2.227 | ESI+ |
| 206 | Carbadox | 263.074 | 178.9 | 1.5 | 0.017 | 2.226 | ESI+ |
| 207 | 4-(4-Iodophenyl)-1,3-thiazol-2-ylamine | 302.946 | 281.5 | 1.5 | 0.006 | 2.218 | ESI+ |
| 208 | SM(d18:1/17:0) | 717.590 | 104.2 | 2.0 | 0.000 | 2.202 | ESI+ |
| 209 | 1,2-Dipalmitoleoyl-sn-glycero-3-phosphocholine | 730.537 | 36.6 | 1.9 | 0.000 | 2.200 | ESI+ |
| 210 | (5-Bromo-3-pyridyl)(4-pyrrolidinopiperidino)methanone | 338.087 | 189.3 | 1.1 | 0.038 | 2.195 | ESI+ |
| 211 | 1-Hydroxy-2-naphthoic acid | 187.042 | 118 | 2.1 | 0.000 | 2.189 | ESI- |
| 212 | N-Acetyl-D-glucosamine | 204.087 | 87.1 | 2.0 | 0.001 | 2.188 | ESI+ |
| 213 | (R)-S-Lactoylglutathione | 416.053 | 215.2 | 1.6 | 0.004 | 2.185 | ESI- |
| 214 | PERILLIC ACID (-) | 165.092 | 26.3 | 1.5 | 0.017 | 2.180 | ESI- |

Continuation Table S3

| No. | Metabolite | Retention time (s) | Ion (m/z) | VIP | P-value | Fold change | ESI mode |
| --- | --- | --- | --- | --- | --- | --- | --- |
| 215 | Dexrazoxane | 267.110 | 195.8 | 1.9 | 0.003 | 2.180 | ESI- |
| 216 | PE(P-36:4) | 724.528 | 34.6 | 2.1 | 0.000 | 2.176 | ESI+ |
| 217 | Glu-Gly-Glu | 332.111 | 136.9 | 2.1 | 0.000 | 2.160 | ESI- |
| 218 | 2,2'-(4-(2-Hydroxyethylamino)-3-nitrophenylazanediyl)diethanol | 286.140 | 241.5 | 1.3 | 0.010 | 2.154 | ESI+ |
| 219 | Cholesteryl sulfate | 465.305 | 13.1 | 1.8 | 0.000 | 2.149 | ESI- |
| 220 | Heptadecanoyl_carnitine | 414.358 | 91.4 | 2.0 | 0.000 | 2.142 | ESI+ |
| 221 | PC(12:0/12:0) | 604.434 | 111.7 | 1.8 | 0.000 | 2.141 | ESI+ |
| 222 | Gulono-1,4-lactone | 177.041 | 55.7 | 1.7 | 0.001 | 2.136 | ESI- |
| 223 | Isodesmosine | 526.293 | 119.2 | 1.8 | 0.001 | 2.134 | ESI+ |
| 224 | PC(38:4) | 810.600 | 90.4 | 1.9 | 0.001 | 2.130 | ESI+ |
| 225 | PE(P-18:1(9Z)/16:0) | 702.544 | 37.3 | 2.2 | 0.000 | 2.125 | ESI+ |
| 226 | 1-Myristoyl-sn-glycero-3-phosphocholine (LPC(14:0/0:0)) | 468.308 | 124.2 | 1.9 | 0.000 | 2.123 | ESI+ |
| 227 | Xanthurenic acid | 206.045 | 89 | 1.6 | 0.011 | 2.106 | ESI+ |
| 228 | Isocaproic acid | 115.077 | 31 | 1.9 | 0.000 | 2.106 | ESI- |
| 229 | Caproic acid | 115.077 | 31 | 1.9 | 0.000 | 2.106 | ESI- |
| 230 | Carnosine | 227.114 | 245.4 | 1.2 | 0.028 | 2.104 | ESI+ |
| 231 | 4-(Hydroxymethyl)benzenediazonium(1+) | 136.062 | 129.3 | 1.7 | 0.007 | 2.100 | ESI+ |
| 232 | Tetradecenedioylcarnitine (Car(14:1-O2)) | 400.270 | 182.2 | 1.4 | 0.022 | 2.088 | ESI+ |
| 233 | N-[4-(Pyridin-2-yl)-1,3-thiazol-2-yl]cyclopentanecarboxamide | 272.087 | 220.2 | 1.7 | 0.004 | 2.081 | ESI- |
| 234 | 1-Stearoyl-2-arachidonoyl-sn-glycero-3-phospho-(1'-myo-inositol) | 885.551 | 123.9 | 2.0 | 0.001 | 2.072 | ESI- |
| 235 | Androstan-4,6-diene-17.beta.-ol-3-one | 287.197 | 161.2 | 1.7 | 0.007 | 2.064 | ESI+ |
| 236 | PC(P-18:1(9Z)/0:0) | 506.360 | 123.3 | 1.9 | 0.002 | 2.062 | ESI+ |
| 237 | 3-(3-Methoxybenzyl)piperidine | 206.152 | 277.3 | 1.3 | 0.025 | 2.061 | ESI+ |
| 238 | 1-[1-(1-Methylcyclooctyl)-4-piperidinyl]-2-[(3R)-3-piperidinyl]-1H-benzimidazole | 409.331 | 28 | 1.4 | 0.026 | 2.054 | ESI+ |
| 239 | LPC(18:2/0:0) | 520.340 | 119.1 | 2.0 | 0.000 | 2.052 | ESI+ |

Continuation Table S3

| No. | Metabolite | Retention time (s) | Ion (m/z) | VIP | P-value | Fold change | ESI mode |
| --- | --- | --- | --- | --- | --- | --- | --- |
| 240 | Tegaserod | 302.197 | 174.1 | 1.5 | 0.010 | 2.051 | ESI+ |
| 241 | Allopurinol-1-ribonucleoside | 251.078 | 43.9 | 1.3 | 0.015 | 2.043 | ESI+ |
| 242 | N-Acetyl-2-carboxybenzenesulfonamide | 242.013 | 15.1 | 1.2 | 0.030 | 2.042 | ESI- |
| 243 | Trigonelline | 138.055 | 171.4 | 1.6 | 0.003 | 2.036 | ESI+ |
| 244 | 3-Pyridylacetic acid | 138.055 | 171.4 | 1.6 | 0.003 | 2.036 | ESI+ |
| 245 | M171T139 | 171.028 | 139.1 | 2.1 | 0.000 | 2.034 | ESI- |
| 246 | Pyrophosphate | 176.936 | 281.7 | 1.9 | 0.001 | 2.030 | ESI- |
| 247 | 1-(1Z-Octadecenyl)-2-(5Z,8Z,11Z,14Z-eicosatetraenoyl)-sn-glycero-3-phosphoethanolamine | 752.559 | 34 | 2.2 | 0.000 | 2.015 | ESI+ |
| 248 | Acetylpyruvate | 111.009 | 91.7 | 1.2 | 0.022 | 2.014 | ESI- |
| 249 | Persicachrome | 385.278 | 128.7 | 1.7 | 0.000 | 2.011 | ESI+ |
| 250 | Lys-Val | 246.182 | 312.4 | 1.9 | 0.002 | 2.010 | ESI+ |
| 251 | Nicotinamide N-oxide | 139.050 | 60.8 | 1.3 | 0.040 | 2.010 | ESI+ |
| 252 | 1,3-Dihydroxyacetone_dimer | 179.056 | 117.4 | 2.2 | 0.000 | 2.006 | ESI- |
| 253 | PC(22:4(7Z,10Z,13Z,16Z)/20:4(5Z,8Z,11Z,14Z)) | 858.598 | 32.7 | 1.7 | 0.002 | 1.999 | ESI+ |
| 254 | PC(42:2) | 870.691 | 33.1 | 1.9 | 0.000 | 1.995 | ESI+ |
| 255 | Uracil | 111.020 | 33.6 | 2.0 | 0.000 | 1.994 | ESI- |
| 256 | (2-aminoethoxy)[2-[hexadec-9-enoyloxy]-3-[octadeca-1.11-dien-1-yloxy]propoxy]phosphinic acid | 698.515 | 36.9 | 1.7 | 0.003 | 1.993 | ESI- |
| 257 | Oxopalmitoleylcarnitine (Car(16:2-O)) | 412.306 | 125.3 | 1.8 | 0.000 | 1.989 | ESI+ |
| 258 | 5-Aminolevulinic acid | 130.051 | 205.1 | 1.9 | 0.000 | 1.988 | ESI- |
| 259 | 4-Hydroxyproline | 130.051 | 205.1 | 1.9 | 0.000 | 1.988 | ESI- |
| 260 | Kifunensine | 233.079 | 26.5 | 1.7 | 0.004 | 1.982 | ESI+ |
| 261 | M375T88 | 375.185 | 88.2 | 1.8 | 0.000 | 1.980 | ESI- |
| 262 | [(7-Oxo-7H-benzo[de]anthracen-3-yl)sulfanyl]acetic acid | 321.060 | 53.9 | 1.8 | 0.000 | 1.977 | ESI+ |
| 263 | Oxotetradecenoylcarnitine (Car(14:2-O)) | 384.275 | 128.6 | 1.8 | 0.001 | 1.977 | ESI+ |
| 264 | N-Acetyl-D-galactosamine 4-sulfate | 300.040 | 87.7 | 2.0 | 0.000 | 1.974 | ESI- |

Continuation Table S3

| No. | Metabolite | Retention time (s) | Ion (m/z) | VIP | P-value | Fold change | ESI mode |
| --- | --- | --- | --- | --- | --- | --- | --- |
| 265 | 9,10-Dihydrojasmonic acid | 211.134 | 37.5 | 1.5 | 0.007 | 1.973 | ESI- |
| 266 | 3-(.alpha.-L-Arabinofuranosyloxy)pimara-8(14),15-dien-12-yl 6-deoxy-.beta.-D-galactopyranoside | 581.335 | 141.9 | 1.7 | 0.006 | 1.972 | ESI- |
| 267 | all-trans-5,6-Epoxyretinoic acid | 315.197 | 38.8 | 1.2 | 0.036 | 1.970 | ESI- |
| 268 | M527T279 | 526.999 | 279.4 | 1.4 | 0.024 | 1.969 | ESI+ |
| 269 | SM(d16:1/24:1(15Z)) | 785.652 | 26.3 | 1.6 | 0.023 | 1.967 | ESI+ |
| 270 | Gaylussacin | 417.120 | 125.3 | 1.7 | 0.001 | 1.963 | ESI- |
| 271 | 4,5-Epoxy-7Z,10Z,13Z,16Z,19Z-docosapentaenoic acid, methyl ester | 359.263 | 133.2 | 1.6 | 0.011 | 1.948 | ESI+ |
| 272 | 3-Nitroaniline | 139.050 | 46.5 | 1.4 | 0.020 | 1.945 | ESI+ |
| 273 | PRI_260.1759_12.9 | 260.172 | 299.1 | 1.7 | 0.004 | 1.943 | ESI+ |
| 274 | 9-(5-O-Methylpentofuranosyl)-1,9-dihydro-6H-purin-6-one | 283.104 | 56.4 | 1.9 | 0.000 | 1.943 | ESI+ |
| 275 | Eicosapentaenoic acid | 301.218 | 19 | 1.6 | 0.019 | 1.939 | ESI- |
| 276 | Allopurinol | 137.046 | 45.2 | 1.4 | 0.006 | 1.933 | ESI+ |
| 277 | Lactate | 89.025 | 117.4 | 2.2 | 0.000 | 1.930 | ESI- |
| 278 | Ikarugamycin | 479.291 | 48.6 | 1.7 | 0.007 | 1.926 | ESI+ |
| 279 | 2-(Hydroxymethyl)-3-(acetamidomethylene)succinate | 233.078 | 129.3 | 1.5 | 0.016 | 1.921 | ESI- |
| 280 | 1-Palmitoyl-2-thiopalmitoyl phosphatidylcholine | 750.539 | 34 | 2.0 | 0.000 | 1.921 | ESI+ |
| 281 | Nodakenitin | 247.093 | 281.5 | 1.7 | 0.007 | 1.916 | ESI+ |
| 282 | 3-(4-Chlorophenyl)-1H-pyrazole | 177.023 | 26.3 | 1.2 | 0.018 | 1.916 | ESI- |
| 283 | (2-{[3-hydroxy-2-tetradecanamidooctadec-4-en-1-yl phosphonato]oxy}ethyl)trimethylazanium | 733.552 | 107.4 | 1.8 | 0.001 | 1.912 | ESI- |
| 284 | PC(18:0/14:0) | 734.569 | 36.6 | 2.2 | 0.000 | 1.912 | ESI+ |
| 285 | N-Acetylalanine | 130.051 | 139.1 | 1.2 | 0.041 | 1.912 | ESI- |
| 286 | 4-Oxo-4-((3-oxodecan-2-yl)amino)butanoic acid | 272.186 | 138.3 | 1.8 | 0.000 | 1.906 | ESI+ |

Continuation Table S3

| No. | Metabolite | Retention time (s) | Ion (m/z) | VIP | P-value | Fold change | ESI mode |
| --- | --- | --- | --- | --- | --- | --- | --- |
| 287 | Malonyl-L-carnitine | 248.113 | 226.5 | 1.2 | 0.037 | 1.905 | ESI+ |
| 288 | S1P(d18:1) | 380.256 | 183.5 | 1.5 | 0.014 | 1.900 | ESI+ |
| 289 | 1-Methylnicotinamide | 137.071 | 190 | 1.2 | 0.021 | 1.899 | ESI+ |
| 290 | 1-Behenoyl-2-hydroxy-sn-glycero-3-phosphocholine | 580.434 | 111.6 | 2.0 | 0.000 | 1.898 | ESI+ |
| 291 | PC(20:2(11Z,14Z)/18:1(9Z)) | 812.620 | 91.3 | 1.7 | 0.001 | 1.894 | ESI+ |
| 292 | N2-Benzyl-1,3,5-triazine-2,4-diamine | 202.108 | 157.3 | 2.0 | 0.000 | 1.888 | ESI+ |
| 293 | N-Propyl-9H-purin-6-amine | 176.093 | 161.4 | 1.6 | 0.011 | 1.887 | ESI- |
| 294 | 5-Carbethoxycytosine | 184.073 | 34.6 | 2.0 | 0.001 | 1.877 | ESI+ |
| 295 | Kynurenic acid | 190.050 | 89.7 | 1.1 | 0.044 | 1.874 | ESI+ |
| 296 | .delta.-Tocotrienol | 397.314 | 98.3 | 1.6 | 0.001 | 1.873 | ESI+ |
| 297 | Xylulose | 149.046 | 54.3 | 1.7 | 0.001 | 1.868 | ESI- |
| 298 | Ribothymidine | 257.078 | 52 | 1.7 | 0.006 | 1.867 | ESI- |
| 299 | PI(18:0/0:0) | 601.336 | 153.1 | 1.7 | 0.004 | 1.866 | ESI+ |
| 300 | [2-(carbamoyloxymethyl)-2-methylpentyl]_N-propan-2-ylcarbamate | 261.181 | 161.3 | 2.0 | 0.000 | 1.864 | ESI+ |
| 301 | Putative Phenylalanine conjugated chenodeoxycholic acid | 540.366 | 130.7 | 1.8 | 0.001 | 1.862 | ESI+ |
| 302 | Altenuene | 291.084 | 288.6 | 1.3 | 0.028 | 1.848 | ESI- |
| 303 | Oxopalmitoylcarnitine (Car(16:1-O)) | 396.311 | 98.5 | 1.6 | 0.001 | 1.845 | ESI+ |
| 304 | Mangiferdesmethylursanone | 429.373 | 15.4 | 1.8 | 0.000 | 1.844 | ESI+ |
| 305 | PC(20:3(8Z,11Z,14Z)/18:2(9Z,12Z)) | 808.583 | 90.8 | 1.7 | 0.000 | 1.838 | ESI+ |
| 306 | Probucol | 515.306 | 23.7 | 1.1 | 0.036 | 1.838 | ESI- |
| 307 | 1-Oleoyl-2-palmitoyl-sn-glycero-3-phosphocholine | 760.582 | 90.3 | 1.8 | 0.006 | 1.837 | ESI+ |
| 308 | 2-Methylguanosine | 298.114 | 102.8 | 1.3 | 0.028 | 1.827 | ESI+ |
| 309 | 1-Methylguanosine | 298.114 | 102.8 | 1.3 | 0.028 | 1.827 | ESI+ |
| 310 | LPA(20:4) | 459.251 | 163.6 | 1.4 | 0.032 | 1.820 | ESI+ |
| 311 | 6-(1-Pyrrolidinyl)-1H-purine | 188.093 | 134.2 | 1.5 | 0.007 | 1.819 | ESI- |

Continuation Table S3

| No. | Metabolite | Retention time (s) | Ion (m/z) | VIP | P-value | Fold change | ESI mode |
| --- | --- | --- | --- | --- | --- | --- | --- |
| 312 | GlcCer(d18:1/16:0) | 700.572 | 26.4 | 1.9 | 0.000 | 1.813 | ESI+ |
| 313 | Prolylhydroxyproline | 229.118 | 244.3 | 2.0 | 0.000 | 1.811 | ESI+ |
| 314 | Uric acid | 167.021 | 193.5 | 1.4 | 0.020 | 1.809 | ESI- |
| 315 | (2-aminoethoxy)[2-[docosa-4.7.10.13.16.19-hexaenoyloxy]-3-[hexadec-1-en-1-yloxy]propoxy]phosphinic acid | 746.515 | 34.9 | 1.9 | 0.001 | 1.808 | ESI- |
| 316 | PC(38:5) | 790.573 | 32.7 | 1.6 | 0.015 | 1.807 | ESI+ |
| 317 | GKK 1032B | 500.279 | 120 | 1.7 | 0.003 | 1.804 | ESI- |
| 318 | PC(18:3(6Z,9Z,12Z)/18:0) | 784.582 | 90 | 1.6 | 0.003 | 1.800 | ESI+ |
| 319 | Car(18:1) | 426.358 | 89.4 | 1.8 | 0.000 | 1.800 | ESI+ |
| 320 | 1-Methylhexahydroazepin-4-one | 128.107 | 20.9 | 1.5 | 0.012 | 1.799 | ESI+ |
| 321 | (3R)-3-Hydroxy-L-proline | 132.065 | 249.4 | 1.6 | 0.013 | 1.798 | ESI+ |
| 322 | Oxotridecanoylcarnitine (Car(13:1-O)) | 372.275 | 134.2 | 1.9 | 0.000 | 1.796 | ESI+ |
| 323 | Fagomine | 148.097 | 178.1 | 1.9 | 0.001 | 1.796 | ESI+ |
| 324 | M211T168 | 211.002 | 168.3 | 1.4 | 0.017 | 1.794 | ESI- |
| 325 | 9-Riburonosyladenine | 318.029 | 157.1 | 1.8 | 0.000 | 1.791 | ESI- |
| 326 | (3beta,22R,23R,24S)-3,22,23-Trihydroxystigmastan-6-one | 463.379 | 15.4 | 2.0 | 0.000 | 1.791 | ESI+ |
| 327 | 1,2-Dioleoyl-sn-glycero-3-phosphoethanolamine-N,N-dimethyl | 772.583 | 18 | 1.7 | 0.003 | 1.787 | ESI+ |
| 328 | Fumaric acid | 115.004 | 230.5 | 1.2 | 0.029 | 1.780 | ESI- |
| 329 | LPE(P-16:0) | 438.297 | 114.6 | 2.0 | 0.000 | 1.780 | ESI+ |
| 330 | 3-Hydroxystigmast-5-en-7-one | 429.377 | 89 | 1.5 | 0.008 | 1.779 | ESI+ |
| 331 | .alpha.-Hydroxymetoprolol | 284.186 | 136.8 | 1.6 | 0.002 | 1.778 | ESI+ |
| 332 | PC(20:2(11Z,14Z)/15:0) | 772.583 | 35.3 | 1.8 | 0.001 | 1.777 | ESI+ |
| 333 | Oleoylcarnitine (Car(18:1)) | 426.358 | 26.8 | 1.4 | 0.023 | 1.767 | ESI+ |
| 334 | 3-(.beta.-D-Glucopyranosyloxy)-15-hydroxy-2,15-dimethylhexadecanoic acid | 477.305 | 13.1 | 1.5 | 0.010 | 1.766 | ESI- |

Continuation Table S3

| No. | Metabolite | Retention time (s) | Ion (m/z) | VIP | P-value | Fold change | ESI mode |
| --- | --- | --- | --- | --- | --- | --- | --- |
| 335 | Pantothenic acid | 218.104 | 157.3 | 1.8 | 0.000 | 1.766 | ESI- |
| 336 | 1-Stearoyl-2-linoleoyl-sn-glycero-3-phospho-(1'-rac-glycerol) | 773.535 | 34.2 | 1.5 | 0.004 | 1.762 | ESI- |
| 337 | N-(3-Dimethylaminopropyl)-N'-ethylcarbodiimide | 156.150 | 181.4 | 1.2 | 0.044 | 1.762 | ESI+ |
| 338 | Glabrone | 359.092 | 219.7 | 1.4 | 0.025 | 1.755 | ESI+ |
| 339 | Cys-Tyr | 283.079 | 144.3 | 1.9 | 0.001 | 1.755 | ESI- |
| 340 | PC(37:4) | 796.583 | 18 | 1.7 | 0.003 | 1.751 | ESI+ |
| 341 | (1R)-4-(3-Hydroxybutyl)-3,5,5-trimethylcyclohex-3-en-1-yl 6-O-.beta.-D-glucopyranosyl-.beta.-D-glucopyranoside | 535.269 | 126.7 | 1.1 | 0.044 | 1.750 | ESI- |
| 342 | (2-aminoethoxy)[2-[octadec-9-enoyloxy]-3-[octadeca-1.11-dien-1-yloxy]propoxy]phosphinic acid | 726.546 | 36.7 | 1.6 | 0.007 | 1.747 | ESI- |
| 343 | 2-(Hydroxymethyl)-6-methylpyridin-3-ol | 138.056 | 102.8 | 2.0 | 0.000 | 1.746 | ESI- |
| 344 | 1-Lignoceroyl-2-hydroxy-sn-glycero-3-phosphocholine | 608.465 | 108.8 | 1.9 | 0.000 | 1.746 | ESI+ |
| 345 | 1-(2-Thienyl)-1-heptanone | 197.101 | 321.3 | 1.5 | 0.003 | 1.745 | ESI+ |
| 346 | LPC(15:0) | 482.324 | 122.1 | 1.8 | 0.000 | 1.745 | ESI+ |
| 347 | 3'-O-Methylguanosine | 296.101 | 103.5 | 1.7 | 0.005 | 1.744 | ESI- |
| 348 | 2'-O-Methylguanosine | 296.101 | 103.5 | 1.7 | 0.005 | 1.744 | ESI- |
| 349 | 2,4-Bis(trifluoromethyl)pyrimidine-5-carboxylic acid | 258.992 | 87.6 | 1.3 | 0.033 | 1.739 | ESI- |
| 350 | Hispidulin 7-glucuronide | 475.089 | 87.6 | 1.2 | 0.038 | 1.735 | ESI- |
| 351 | 2-Chloro-N,N-dimethylpropan-1-amine | 122.071 | 210.3 | 1.7 | 0.002 | 1.735 | ESI+ |
| 352 | Hydroxyphenyllactic acid | 181.051 | 90.9 | 1.6 | 0.001 | 1.733 | ESI- |
| 353 | Thiamine | 265.112 | 210.3 | 1.7 | 0.001 | 1.733 | ESI+ |
| 354 | 2-Chloroticlopidine | 298.025 | 215 | 1.7 | 0.001 | 1.731 | ESI+ |
| 355 | Pro-Leu | 229.155 | 181.4 | 1.6 | 0.012 | 1.730 | ESI+ |

Continuation Table S3

| No. | Metabolite | Retention time (s) | Ion (m/z) | VIP | P-value | Fold change | ESI mode |
| --- | --- | --- | --- | --- | --- | --- | --- |
| 356 | 6-Benzyl-3-butan-2-yl-9-(7,8-dihydroxy-6-oxooctyl)-1,4,7,10-tetrazabicyclo[10.4.0]hexadecane-2,5,8,11-tetrone | 585.331 | 108.1 | 1.5 | 0.013 | 1.727 | ESI- |
| 357 | SM(d18:0/16:0) | 705.580 | 105.7 | 1.9 | 0.003 | 1.725 | ESI+ |
| 358 | 4-(1-Benzofuran-2-yl)pyridine | 196.073 | 156.3 | 1.5 | 0.018 | 1.724 | ESI+ |
| 359 | Steviol | 319.227 | 16 | 1.8 | 0.003 | 1.723 | ESI+ |
| 360 | N6,N6,N6-Trimethyllysine | 189.160 | 303.8 | 2.1 | 0.000 | 1.718 | ESI+ |
| 361 | PC(39:6) | 820.582 | 33.7 | 1.6 | 0.007 | 1.717 | ESI+ |
| 362 | Hypotaurine | 108.013 | 203.9 | 1.4 | 0.020 | 1.715 | ESI- |
| 363 | LPC(17:0/0:0) | 510.355 | 118.7 | 1.9 | 0.000 | 1.710 | ESI+ |
| 364 | N-Acetylmannosamine | 204.087 | 142.3 | 1.6 | 0.005 | 1.709 | ESI+ |
| 365 | N-Acetylgalactosamine | 204.087 | 142.3 | 1.6 | 0.005 | 1.709 | ESI+ |
| 366 | 4-Amino-2-oxo-1,2-dihydropyrimidine-5-carboxylic acid | 154.026 | 48.1 | 1.4 | 0.007 | 1.707 | ESI- |
| 367 | Nitrofurazone | 197.032 | 48.1 | 1.4 | 0.007 | 1.705 | ESI- |
| 368 | 2-Amino-6-sulfanyl-3,5-pyridinedicarbonitrile | 177.025 | 221.6 | 1.8 | 0.001 | 1.701 | ESI+ |
| 369 | Beta-Ecdysterone | 481.311 | 122.7 | 1.6 | 0.001 | 1.694 | ESI+ |
| 370 | N-Acetyl-L-Prolinamide | 157.097 | 28.2 | 1.9 | 0.000 | 1.687 | ESI+ |
| 371 | Arsenobetaine | 179.005 | 166.9 | 1.5 | 0.002 | 1.686 | ESI+ |
| 372 | (2R)-3-Hydroxyisovaleroylcarnitine | 262.165 | 184.9 | 1.6 | 0.005 | 1.680 | ESI+ |
| 373 | Car(18:0) | 428.374 | 89 | 1.5 | 0.010 | 1.679 | ESI+ |
| 374 | trans-Hexadec-2-enoyl_carnitine | 398.326 | 96.1 | 1.6 | 0.006 | 1.679 | ESI+ |
| 375 | N-(4-Ethoxyphenyl)-1,3-benzothiazol-2-amine | 271.092 | 91.7 | 1.3 | 0.029 | 1.679 | ESI+ |
| 376 | 2-Amino-5,5,5-trifluoropentanoic acid | 170.044 | 213.6 | 1.2 | 0.028 | 1.673 | ESI- |
| 377 | Pipecolamide | 129.102 | 125.3 | 1.9 | 0.000 | 1.668 | ESI+ |
| 378 | Arecaidine | 142.086 | 152.8 | 1.8 | 0.000 | 1.658 | ESI+ |
| 379 | 35S-Methylokadaic_acid_7-hexadecanoate | 1057.715 | 117.3 | 1.5 | 0.012 | 1.657 | ESI+ |

Continuation Table S3

| No. | Metabolite | Retention time (s) | Ion (m/z) | VIP | P-value | Fold change | ESI mode |
| --- | --- | --- | --- | --- | --- | --- | --- |
| 380 | 1,2-Distearoyl-sn-glycero-3-phospho-(1'-rac-glycerol) | 777.564 | 34.9 | 1.7 | 0.001 | 1.654 | ESI- |
| 381 | HEPTANOIC ACID | 129.092 | 27.6 | 2.0 | 0.001 | 1.653 | ESI- |
| 382 | O-Acetylserine | 146.046 | 173.3 | 1.6 | 0.004 | 1.653 | ESI- |
| 383 | Hippuric acid | 178.051 | 95.6 | 1.3 | 0.019 | 1.650 | ESI- |
| 384 | PC(16:0/16:0) | 734.569 | 18.1 | 1.9 | 0.002 | 1.649 | ESI+ |
| 385 | 3-Methyladenine | 150.078 | 137.1 | 1.6 | 0.005 | 1.648 | ESI+ |
| 386 | 7-Hydroxy-4-(trifluoromethyl)coumarin | 229.012 | 253.3 | 1.4 | 0.022 | 1.646 | ESI- |
| 387 | 3-Phenyl-N-(phenylacetyl)-.beta.-alanine | 282.112 | 210.1 | 2.0 | 0.000 | 1.646 | ESI- |
| 388 | Dimethylglycine | 104.071 | 179.3 | 1.7 | 0.001 | 1.645 | ESI+ |
| 389 | .alpha.-Tocopherol nicotinate | 536.407 | 103 | 1.6 | 0.005 | 1.644 | ESI+ |
| 390 | 3'-O-Methylinosine | 281.090 | 54.6 | 1.8 | 0.001 | 1.643 | ESI- |
| 391 | 2'-O-Methylinosine | 281.090 | 54.6 | 1.8 | 0.001 | 1.643 | ESI- |
| 392 | PC(35:5) | 766.537 | 47.2 | 1.3 | 0.037 | 1.642 | ESI+ |
| 393 | Erythronolactone | 117.019 | 110 | 1.0 | 0.045 | 1.640 | ESI- |
| 394 | Sakacin_P | 217.118 | 170.8 | 1.5 | 0.008 | 1.637 | ESI+ |
| 395 | Arabinono-1,4-lactone | 147.030 | 33 | 1.4 | 0.029 | 1.636 | ESI- |
| 396 | Sarcosine | 90.055 | 205.4 | 2.0 | 0.000 | 1.630 | ESI+ |
| 397 | Alanine | 90.055 | 205.4 | 2.0 | 0.000 | 1.630 | ESI+ |
| 398 | PC(22:0/22:4(7Z,10Z,13Z,16Z)) | 894.693 | 32 | 1.1 | 0.019 | 1.628 | ESI+ |
| 399 | Myristoylcarnitine | 372.311 | 101.2 | 1.6 | 0.007 | 1.628 | ESI+ |
| 400 | Decamethylcyclopentasiloxane | 371.102 | 15.4 | 1.1 | 0.048 | 1.627 | ESI+ |
| 401 | Indole-3-pyruvic acid | 202.051 | 27.6 | 1.5 | 0.005 | 1.623 | ESI- |
| 402 | 2,4-dihydroxyheptadec-16-ynyl acetate | 325.239 | 48.2 | 1.5 | 0.009 | 1.622 | ESI- |
| 403 | Falimint | 239.100 | 170.8 | 1.3 | 0.013 | 1.622 | ESI+ |
| 404 | 6-Hydroxy-3-succinoylpyridine | 216.025 | 95.5 | 1.2 | 0.043 | 1.619 | ESI- |
| 405 | Eplerenone hydroxy acid | 431.212 | 25 | 1.2 | 0.035 | 1.619 | ESI- |
| 406 | Palmitoyl sphingomyelin | 703.573 | 105.7 | 1.8 | 0.002 | 1.617 | ESI+ |
| 407 | Phenylpyruvic acid | 163.040 | 24.7 | 1.6 | 0.004 | 1.616 | ESI- |

Continuation Table S3

| No. | Metabolite | Retention time (s) | Ion (m/z) | VIP | P-value | Fold change | ESI mode |
| --- | --- | --- | --- | --- | --- | --- | --- |
| 408 | (6E)-8-Methyl-6-nonenoic acid | 169.124 | 24.3 | 1.5 | 0.009 | 1.613 | ESI- |
| 409 | 4-Fluorobenzenesulfonamide | 174.002 | 139.9 | 2.2 | 0.000 | 1.613 | ESI- |
| 410 | PC(18:3(6Z,9Z,12Z)/15:0) | 742.535 | 37.3 | 1.3 | 0.022 | 1.613 | ESI+ |
| 411 | SM(d18:0/18:0) | 733.612 | 103 | 1.8 | 0.000 | 1.612 | ESI+ |
| 412 | cis-4,10,13,16-Docosatetraenoic acid | 331.265 | 19 | 1.7 | 0.003 | 1.612 | ESI- |
| 413 | N,N-Dimethylarginine (ADMA) | 203.150 | 289.9 | 1.7 | 0.003 | 1.611 | ESI+ |
| 414 | SM(d18:1/18:0) | 731.605 | 103 | 1.9 | 0.000 | 1.608 | ESI+ |
| 415 | PC(15:0/18:0) | 748.582 | 36.6 | 1.6 | 0.003 | 1.607 | ESI+ |
| 416 | cis-11.14-Eicosadienoic acid | 307.265 | 19 | 2.0 | 0.000 | 1.607 | ESI- |
| 417 | 2-Oxo-S-phenyl-2-(piperidin-1-yl)ethane-1-sulfonamido | 281.100 | 157.1 | 1.7 | 0.001 | 1.606 | ESI- |
| 418 | Phenacetin | 180.100 | 156.2 | 1.4 | 0.033 | 1.604 | ESI+ |
| 419 | Glucuronic acid | 193.036 | 221.5 | 1.3 | 0.024 | 1.600 | ESI- |
| 420 | 1-(1Z-Octadecenyl)-2-(4Z,7Z,10Z,13Z,16Z,19Z-docosahexaenoyl)-sn-glycero-3-phosphoethanolamine | 774.546 | 34.3 | 1.7 | 0.001 | 1.600 | ESI- |
| 421 | 1,7-Dioxa-4,10-diazacyclododecane | 175.144 | 309.2 | 1.9 | 0.000 | 1.599 | ESI+ |
| 422 | 4,4'-Sulfonylbisphenol | 249.022 | 175.4 | 1.1 | 0.025 | 1.598 | ESI- |
| 423 | Diglycine | 131.047 | 221.5 | 1.8 | 0.002 | 1.598 | ESI- |
| 424 | 6-(Chloromethyl)-1,3,5-triazine-2,4-diamine | 160.037 | 171.4 | 1.3 | 0.039 | 1.594 | ESI+ |
| 425 | beta-Alanine | 88.041 | 205.3 | 1.9 | 0.000 | 1.591 | ESI- |
| 426 | D-Saccharic acid 1,4-lactone | 191.020 | 47.7 | 1.5 | 0.004 | 1.587 | ESI- |
| 427 | Histidine | 156.077 | 228.6 | 1.6 | 0.018 | 1.587 | ESI+ |
| 428 | Hexenoylcarnitine (Car(6:1)) | 258.170 | 145 | 1.5 | 0.005 | 1.587 | ESI+ |
| 429 | 5-(Tetradecyloxy)-2-furoic acid | 323.223 | 55.9 | 1.3 | 0.013 | 1.586 | ESI- |
| 430 | PC(37:2) | 800.614 | 18.1 | 1.8 | 0.001 | 1.583 | ESI+ |

Continuation Table S3

| No. | Metabolite | Retention time (s) | Ion (m/z) | VIP | P-value | Fold change | ESI mode |
| --- | --- | --- | --- | --- | --- | --- | --- |
| 431 | Indole-3-carboxaldehyde | 144.046 | 17.7 | 1.5 | 0.013 | 1.578 | ESI- |
| 432 | 5-Oxo-L-prolyl-L-proline | 225.088 | 169.9 | 1.5 | 0.005 | 1.577 | ESI- |
| 433 | PC(22:2) | 572.371 | 114.1 | 1.5 | 0.008 | 1.577 | ESI+ |
| 434 | 2-(Butan-2-yl)-5a-hydroxy-4-(4-oxoquinazolin-3(4H)-yl)-4,5,5a,9c-tetrahydro-3H-2a,9b-diazacyclopenta[jk]fluorene-1,3(2H)-dione | 443.176 | 25 | 1.2 | 0.030 | 1.577 | ESI- |
| 435 | Phosphate | 96.970 | 281.7 | 2.0 | 0.000 | 1.572 | ESI- |
| 436 | Tauropine | 213.050 | 220.8 | 1.8 | 0.001 | 1.571 | ESI- |
| 437 | Stachydrine | 144.102 | 161.2 | 1.7 | 0.002 | 1.571 | ESI+ |
| 438 | 1-Allylpiperazine | 127.123 | 179 | 1.4 | 0.026 | 1.569 | ESI+ |
| 439 | 2-(2,4-Dichlorophenyl)-3-[4-(dimethylamino)phenyl]acrylonitrile | 317.057 | 5.1 | 1.4 | 0.007 | 1.567 | ESI+ |
| 440 | PC(15:0/20:1(11Z)) | 774.595 | 35.3 | 1.5 | 0.014 | 1.566 | ESI+ |
| 441 | Porphobilinogen | 249.085 | 170.1 | 1.2 | 0.036 | 1.566 | ESI+ |
| 442 | 2-Hydroxymyristic_Acid | 243.197 | 26.7 | 1.4 | 0.016 | 1.566 | ESI- |
| 443 | 5-(4-Chlorophenyl)pyrazine-2,3-dicarbonitrile | 241.031 | 279.3 | 1.1 | 0.030 | 1.565 | ESI+ |
| 444 | 1-oleoyl-2-myristoyl-sn-glycero-3-phosphocholine | 732.553 | 36.6 | 1.5 | 0.035 | 1.564 | ESI+ |
| 445 | Alfacalcidol | 383.331 | 15.7 | 1.8 | 0.001 | 1.555 | ESI+ |
| 446 | 7-Ketocholesterol | 383.331 | 15.7 | 1.8 | 0.001 | 1.555 | ESI+ |
| 447 | PC(15:0/16:0) | 720.553 | 37.3 | 1.5 | 0.008 | 1.552 | ESI+ |
| 448 | beta-Alaninamide | 71.061 | 188.4 | 1.3 | 0.006 | 1.551 | ESI+ |
| 449 | PC(18:1(11Z)/15:0) | 746.566 | 36.6 | 1.3 | 0.034 | 1.550 | ESI+ |
| 450 | 2,4-Dinitrophenol | 183.007 | 31.5 | 1.3 | 0.028 | 1.549 | ESI- |
| 451 | M87T117 | 87.009 | 117.4 | 1.8 | 0.001 | 1.548 | ESI- |
| 452 | 2-Acetylthiazole | 128.018 | 175.4 | 1.8 | 0.000 | 1.544 | ESI+ |
| 453 | 5-Azacytidine | 243.076 | 199.4 | 1.1 | 0.023 | 1.542 | ESI- |
| 454 | M140T161 | 140.030 | 160.6 | 1.5 | 0.002 | 1.536 | ESI- |
| 455 | Octenoylcarnitine (Car(8:1)) | 286.202 | 131.5 | 1.3 | 0.025 | 1.535 | ESI+ |
| 456 | Isocytosine | 112.051 | 137.1 | 1.9 | 0.000 | 1.534 | ESI+ |
| 457 | 9,10-Anthracenedione, 3-(1,2-dihydroxypropyl)-1,6,8-trihydroxy- | 329.071 | 38.1 | 1.3 | 0.035 | 1.534 | ESI- |

Continuation Table S3

| No. | Metabolite | Retention time (s) | Ion (m/z) | VIP | P-value | Fold change | ESI mode |
| --- | --- | --- | --- | --- | --- | --- | --- |
| 458 | (2E,6E,12E)-19-(2-Amino-2-oxoethyl)-9,11-dihydroxy-8-methoxy-10,12,14-trimethyl-15-oxohenicosa-2,6,12-trienedioic acid | 524.279 | 119 | 1.6 | 0.006 | 1.533 | ESI- |
| 459 | N,N-Dimethyl-L-valine | 146.118 | 223.6 | 1.8 | 0.001 | 1.530 | ESI+ |
| 460 | PC(18:0/16:0) | 744.591 | 35.3 | 2.0 | 0.000 | 1.527 | ESI+ |
| 461 | PC(17:0/17:0) | 744.591 | 35.3 | 2.0 | 0.000 | 1.527 | ESI+ |
| 462 | PC(16:0/18:0) | 744.591 | 35.3 | 2.0 | 0.000 | 1.527 | ESI+ |
| 463 | LPC(20:0) | 552.402 | 114.1 | 1.4 | 0.011 | 1.525 | ESI+ |
| 464 | 2-Cyano-N-(3,4-difluorophenyl)acetamide | 197.053 | 178.5 | 1.5 | 0.016 | 1.524 | ESI+ |
| 465 | PC(18:2(9Z,12Z)/P-16:0) | 742.576 | 34.7 | 2.1 | 0.000 | 1.523 | ESI+ |
| 466 | 4-Methoxy-2,4-dioxobutanoic acid | 145.015 | 54.5 | 1.4 | 0.017 | 1.521 | ESI- |
| 467 | 1-O-Hexadecyl-sn-glycero-3-phosphocholine (LPC(O-16:0/0:0)) | 482.360 | 124.2 | 1.8 | 0.001 | 1.519 | ESI+ |
| 468 | PC(20:3(5Z,8Z,11Z)/15:0) | 770.564 | 36 | 1.6 | 0.008 | 1.518 | ESI+ |
| 469 | PC(20:0/20:3(8Z,11Z,14Z)) | 840.646 | 39.9 | 1.4 | 0.036 | 1.514 | ESI+ |
| 470 | 3'-O-methylcytidine | 258.109 | 76.9 | 2.0 | 0.000 | 1.511 | ESI+ |
| 471 | 2'-O-methylcytidine | 258.109 | 76.9 | 2.0 | 0.000 | 1.511 | ESI+ |
| 472 | PC(20:4(8Z,11Z,14Z,17Z)/15:0) | 768.552 | 35.3 | 1.5 | 0.012 | 1.508 | ESI+ |
| 473 | Heptanedioic acid, 1-(2-cyclopentylidenehydrazide) | 241.155 | 182.9 | 1.7 | 0.002 | 1.507 | ESI+ |
| 474 | Cytidine | 244.093 | 137.5 | 1.8 | 0.000 | 1.507 | ESI+ |
| 475 | Acetyl-CoA | 810.138 | 235.5 | 1.3 | 0.027 | 1.501 | ESI+ |
| 476 | 2,3,4-Pentanetrione, 3-[(4-nitrophenyl)hydrazone] | 250.080 | 111.4 | 1.9 | 0.001 | 0.666 | ESI+ |
| 477 | 5,5-Dimethyl-3-(4-nitrobenzyl)-2,4-imidazolidinedione | 264.095 | 101.7 | 1.2 | 0.024 | 0.664 | ESI+ |
| 478 | 1-Nitrosonaphthalene | 158.060 | 24.1 | 1.2 | 0.039 | 0.662 | ESI+ |
| 479 | 4-Methylquinolin-2-ol | 158.061 | 17.1 | 1.3 | 0.032 | 0.661 | ESI- |
| 480 | alpha-Ketoisovaleric acid | 115.040 | 27.6 | 1.4 | 0.015 | 0.660 | ESI- |

Continuation Table S3

| No. | Metabolite | Retention time (s) | Ion (m/z) | VIP | P-value | Fold change | ESI mode |
| --- | --- | --- | --- | --- | --- | --- | --- |
| 481 | Cidofovir | 280.069 | 102.1 | 1.3 | 0.017 | 0.631 | ESI+ |
| 482 | 5-Amino-1-naphthol | 158.061 | 52.9 | 1.0 | 0.027 | 0.628 | ESI- |
| 483 | 2-(3,5-Dihydroxy-4-methoxyphenyl)-5,7-dihydroxy-4-oxo-4H-chromen-3-yl .beta.-D-glucopyranoside | 493.099 | 165.1 | 1.4 | 0.013 | 0.620 | ESI- |
| 484 | cis-4-Hydroxycyclohexanecarboxylic acid | 143.072 | 47.4 | 1.3 | 0.031 | 0.594 | ESI- |
| 485 | (3-Carboxypropyl)trimethylammonium cation | 146.118 | 142.8 | 1.5 | 0.050 | 0.591 | ESI+ |
| 486 | Acetylglycine | 116.036 | 162.7 | 1.5 | 0.012 | 0.591 | ESI- |
| 487 | Hexadecanedioic acid, 3,3,14,14-tetramethyl- | 341.270 | 44.6 | 1.3 | 0.035 | 0.578 | ESI- |
| 488 | Hexadecanedioic acid | 285.208 | 92.9 | 1.4 | 0.027 | 0.555 | ESI- |
| 489 | 4-Hydroxyphenylacetic acid | 151.040 | 98.1 | 1.6 | 0.009 | 0.549 | ESI- |
| 490 | 3-Hydroxyphenylacetic acid | 151.040 | 98.1 | 1.6 | 0.009 | 0.549 | ESI- |
| 491 | M452T166 | 452.083 | 165.7 | 1.6 | 0.004 | 0.549 | ESI- |
| 492 | 5a-Hydroxy-4-(4-oxoquinazolin-3(4H)-yl)-2-(propan-2-yl)-4,5,5a,9c-tetrahydro-3H-2a,9b-diazacyclopenta[jk]fluorene-1,3(2H)-dione | 429.155 | 159.3 | 1.3 | 0.049 | 0.533 | ESI- |
| 493 | 9-HPODE | 311.223 | 53.5 | 1.4 | 0.030 | 0.531 | ESI- |
| 494 | Tridecenoylcarnitine (Car(13:1)) | 356.280 | 102.8 | 1.2 | 0.037 | 0.527 | ESI+ |
| 495 | Thieno[3,2-b][1]benzothiophene-2-carboxylic acid | 232.976 | 158.1 | 1.5 | 0.021 | 0.521 | ESI- |
| 496 | 3-Chloro-5-methylbenzene-1,2-diol | 157.008 | 34.2 | 1.4 | 0.027 | 0.519 | ESI- |
| 497 | Destruxin A | 576.332 | 120 | 1.3 | 0.026 | 0.514 | ESI- |
| 498 | 3-Hydroxyphenylaceitic acid sulfate | 230.997 | 121.9 | 1.4 | 0.044 | 0.513 | ESI- |
| 499 | M554T120 | 554.347 | 120 | 1.8 | 0.000 | 0.512 | ESI- |
| 500 | Octadecanedioic acid | 313.239 | 51.5 | 1.6 | 0.013 | 0.503 | ESI- |
| 501 | 1-(2,5-Dimethylphenoxy)-3-(4-morpholinyl)-2-propanol | 266.173 | 40.7 | 1.0 | 0.047 | 0.488 | ESI+ |

Continuation Table S3

| No. | Metabolite | Retention time (s) | Ion (m/z) | VIP | P-value | Fold change | ESI mode |
| --- | --- | --- | --- | --- | --- | --- | --- |
| 502 | M306T172 | 306.034 | 171.6 | 1.2 | 0.050 | 0.487 | ESI- |
| 503 | 3-[(1E,3E)-Hepta-1,3-dienyl]pentanedioic acid | 225.114 | 38.7 | 1.5 | 0.037 | 0.477 | ESI- |
| 504 | Quinolin-6-yl-acetic acid, methyl ester | 202.086 | 94.4 | 1.3 | 0.036 | 0.465 | ESI+ |
| 505 | Prostaglandin B2 (PGB2) | 333.208 | 42.2 | 1.9 | 0.003 | 0.463 | ESI- |
| 506 | Prostaglandin A2 | 333.208 | 42.2 | 1.9 | 0.003 | 0.463 | ESI- |
| 507 | 5-(2-Chlorophenyl)-4-methyl-4H-1,2,4-triazol-3-yl hydrosulfide | 224.003 | 94.2 | 1.4 | 0.017 | 0.459 | ESI- |
| 508 | p-Toluquinone | 121.030 | 37.5 | 1.4 | 0.014 | 0.453 | ESI- |
| 509 | Benzoic acid | 121.030 | 37.5 | 1.4 | 0.014 | 0.453 | ESI- |
| 510 | 4-Hydroxybenzaldehyde | 121.030 | 37.5 | 1.4 | 0.014 | 0.453 | ESI- |
| 511 | 3-hydroxybenzaldehyde | 121.030 | 37.5 | 1.4 | 0.014 | 0.453 | ESI- |
| 512 | 2-Hydroxybenzothiazole | 150.002 | 17.9 | 1.7 | 0.001 | 0.446 | ESI- |
| 513 | 2-Acetylmaslinic acid | 513.360 | 31.6 | 1.5 | 0.013 | 0.441 | ESI- |
| 514 | Aucubin | 343.167 | 95.7 | 1.5 | 0.008 | 0.439 | ESI- |
| 515 | Methyl 1-hydroxy-2-naphthoate | 201.058 | 124.6 | 1.6 | 0.009 | 0.439 | ESI- |
| 516 | Phenol | 93.035 | 13.7 | 1.3 | 0.030 | 0.432 | ESI- |
| 517 | 4-Oxohexanoic acid | 129.056 | 52.9 | 1.3 | 0.039 | 0.425 | ESI- |
| 518 | Phenylsulfate | 172.992 | 13.7 | 1.3 | 0.034 | 0.424 | ESI- |
| 519 | 3beta-Hydroxy-5-cholestenoic acid (3-HCOA) | 415.322 | 23.7 | 1.5 | 0.038 | 0.408 | ESI- |
| 520 | AM580 | 350.175 | 94.2 | 1.2 | 0.013 | 0.404 | ESI- |
| 521 | 3-Hydroxydecanoic acid | 187.134 | 37.5 | 1.3 | 0.025 | 0.404 | ESI- |
| 522 | 10-Hydroxydecanoic acid | 187.134 | 37.5 | 1.3 | 0.025 | 0.404 | ESI- |
| 523 | Olopatadine | 336.160 | 99.4 | 1.4 | 0.034 | 0.382 | ESI- |
| 524 | 2-Methylhippuric acid | 192.067 | 91.6 | 1.3 | 0.033 | 0.370 | ESI- |
| 525 | 2-(2-((Hexopyranosyloxy)methyl)-3-methylcyclopent-2-en-1-yl)-3-hydroxypropanoic acid | 361.151 | 96.2 | 1.0 | 0.040 | 0.364 | ESI- |
| 526 | Phenylacetylglycine | 194.081 | 91.5 | 1.5 | 0.017 | 0.364 | ESI+ |
| 527 | Oxononanoylcarnitine (Car(9:1-O)) | 316.212 | 147.1 | 1.6 | 0.013 | 0.363 | ESI+ |
| 528 | 4-(1,3-Benzothiazol-2-yl)butanoic acid | 222.058 | 51.8 | 1.5 | 0.016 | 0.350 | ESI+ |

Continuation Table S3

| No. | Metabolite | Retention time (s) | Ion (m/z) | VIP | P-value | Fold change | ESI mode |
| --- | --- | --- | --- | --- | --- | --- | --- |
| 529 | 4-Amino-6-methoxy-3-quinolinecarboxylic acid | 219.076 | 99.5 | 1.1 | 0.026 | 0.348 | ESI+ |
| 530 | p-Cresol glucuronide | 283.083 | 121.9 | 1.3 | 0.042 | 0.335 | ESI- |
| 531 | Isobutyrylglycine | 144.067 | 119.3 | 1.4 | 0.032 | 0.333 | ESI- |
| 532 | M274T92_2 | 274.070 | 92.3 | 1.4 | 0.018 | 0.332 | ESI- |
| 533 | 2-Fluoro-4'-hydroxybenzophenone | 215.054 | 123.9 | 1.8 | 0.001 | 0.318 | ESI- |
| 534 | Octafluorocyclobutane | 200.997 | 164.7 | 1.5 | 0.046 | 0.316 | ESI+ |
| 535 | 8-iso-15-keto-PGE2 | 349.202 | 104.1 | 1.7 | 0.009 | 0.313 | ESI- |
| 536 | 1H-Pyrrole-3-propanoic acid, 5-[(1,2-dihydro-2-oxo-3H-indol-3-ylidene)methyl]-2,4-dimethyl- | 309.128 | 31.5 | 1.4 | 0.010 | 0.312 | ESI- |
| 537 | 1-(3-Chlorophenyl)-6,6-dimethyl-1,6-dihydro-1,3,5-triazine-2,4-diamine | 252.102 | 165 | 1.8 | 0.009 | 0.296 | ESI+ |
| 538 | 3-Hydroxybutyric acid | 103.040 | 123.9 | 1.8 | 0.005 | 0.292 | ESI- |
| 539 | Ethyl 6-chloro-4-hydroxyquinoline-3-carboxylate | 250.026 | 91.6 | 1.5 | 0.014 | 0.291 | ESI- |
| 540 | 6-Acetylcodeine | 342.170 | 165.2 | 1.5 | 0.023 | 0.276 | ESI+ |
| 541 | 3-Chloro-6-(3,5-dimethyl-1H-pyrazol-1-yl)pyridazine | 209.059 | 12.7 | 1.5 | 0.016 | 0.259 | ESI+ |
| 542 | Cortisol 21-sulfate | 441.153 | 165 | 1.3 | 0.037 | 0.259 | ESI- |
| 543 | (5-Chloro-3-methyl-4-isoxazolyl)acetic acid | 173.995 | 13.7 | 1.7 | 0.009 | 0.245 | ESI- |
| 544 | N-3-Hydroxyoctanoyl-L-homoserine lactone | 244.154 | 90.3 | 1.3 | 0.045 | 0.233 | ESI+ |
| 545 | Diclofenac | 296.021 | 170.6 | 1.8 | 0.031 | 0.229 | ESI+ |
| 546 | Valproic acid .beta.-D-glucuronide | 319.140 | 87.6 | 1.4 | 0.030 | 0.213 | ESI- |
| 547 | N-[3-hydroxy-1-{[3.4.5-trihydroxy-6-(hydroxymethyl)oxan-2-yl]oxy}octadec-4-en-2-yl]octadecanamide | 726.582 | 98.1 | 1.8 | 0.016 | 0.192 | ESI- |
| 548 | Phenylalanyl-Histidine | 303.142 | 129.9 | 1.6 | 0.006 | 0.191 | ESI+ |
| 549 | Asn-Trp | 317.125 | 88 | 1.6 | 0.028 | 0.165 | ESI- |

Continuation Table S3

| No. | Metabolite | Retention time (s) | Ion (m/z) | VIP | P-value | Fold change | ESI mode |
| --- | --- | --- | --- | --- | --- | --- | --- |
| 550 | Torachrysone_8-(2-apiosylglucoside) | 541.198 | 156.1 | 1.9 | 0.002 | 0.148 | ESI+ |
| 551 | Ethyl 6-methyl-4-(4-methylphenyl)-2-oxo-1,2,3,4-tetrahydro-5-pyrimidinecarboxylate | 275.143 | 125.3 | 1.6 | 0.018 | 0.142 | ESI+ |
| 552 | Euparin | 215.074 | 124 | 1.8 | 0.011 | 0.139 | ESI- |
| 553 | 4-Ethylpiperidine | 114.128 | 129.9 | 1.6 | 0.022 | 0.127 | ESI+ |
| 554 | Totarol | 287.233 | 30.7 | 1.8 | 0.031 | 0.119 | ESI+ |
| 555 | 2-(Chloromethyl)-5-nitro-1H-benzimidazole | 212.020 | 17.8 | 1.9 | 0.003 | 0.115 | ESI+ |
| 556 | Aspochalasin D | 402.270 | 27.2 | 1.3 | 0.014 | 0.094 | ESI+ |
| 557 | Methoxyfenozide | 369.212 | 132 | 1.4 | 0.014 | 0.065 | ESI+ |
| 558 | 2-Methylpiperidine | 100.112 | 152.7 | 1.5 | 0.014 | 0.061 | ESI+ |
| 559 | L-Alaninamide, N-[2-[2-(hydroxyamino)-2-oxoethyl]-4-methyl-1-oxopentyl]-3-methyl-L-valyl-N-(2-aminoethyl)- | 416.285 | 24.7 | 1.6 | 0.012 | 0.053 | ESI+ |
| 560 | 1-Palmitoyllysophosphatidate | 409.237 | 120.6 | 2.1 | 0.000 | 0.052 | ESI- |
| 561 | Corynanthin | 372.223 | 143.9 | 1.4 | 0.013 | 0.048 | ESI+ |
| 562 | Serine-Cholic Acid | 460.312 | 26 | 1.4 | 0.013 | 0.040 | ESI+ |
| 563 | Benz[a]anthracen-1-ol, 8-methoxy-3-methyl- | 289.126 | 143.1 | 1.4 | 0.013 | 0.040 | ESI+ |
| 564 | N,N-Diethyl-2-aminoethanol | 100.112 | 132.2 | 1.5 | 0.012 | 0.029 | ESI+ |
| 565 | PC(P-19:0) | 518.354 | 25.3 | 1.5 | 0.014 | 0.018 | ESI+ |
